# Ovarian cancer mutational processes drive site-specific immune evasion

**DOI:** 10.1038/s41586-022-05496-1

**Published:** 2022-12-14

**Authors:** Ignacio Vázquez-García, Florian Uhlitz, Nicholas Ceglia, Jamie L. P. Lim, Michelle Wu, Neeman Mohibullah, Juliana Niyazov, Arvin Eric B. Ruiz, Kevin M. Boehm, Viktoria Bojilova, Christopher J. Fong, Tyler Funnell, Diljot Grewal, Eliyahu Havasov, Samantha Leung, Arfath Pasha, Druv M. Patel, Maryam Pourmaleki, Nicole Rusk, Hongyu Shi, Rami Vanguri, Marc J. Williams, Allen W. Zhang, Vance Broach, Dennis S. Chi, Arnaud Da Cruz Paula, Ginger J. Gardner, Sarah H. Kim, Matthew Lennon, Kara Long Roche, Yukio Sonoda, Oliver Zivanovic, Ritika Kundra, Agnes Viale, Fatemeh N. Derakhshan, Luke Geneslaw, Shirin Issa Bhaloo, Ana Maroldi, Rahelly Nunez, Fresia Pareja, Anthe Stylianou, Mahsa Vahdatinia, Yonina Bykov, Rachel N. Grisham, Ying L. Liu, Yulia Lakhman, Ines Nikolovski, Daniel Kelly, Jianjiong Gao, Andrea Schietinger, Travis J. Hollmann, Samuel F. Bakhoum, Robert A. Soslow, Lora H. Ellenson, Nadeem R. Abu-Rustum, Carol Aghajanian, Claire F. Friedman, Andrew McPherson, Britta Weigelt, Dmitriy Zamarin, Sohrab P. Shah

**Affiliations:** 1 Computational Oncology, Department of Epidemiology and Biostatistics, Memorial Sloan Kettering Cancer Center, New York, NY USA; 2 Department of Surgery, Memorial Sloan Kettering Cancer Center, New York, NY USA; 3 Integrated Genomics Operation, Memorial Sloan Kettering Cancer Center, New York, NY USA; 4 Department of Pathology and Laboratory Medicine, Memorial Sloan Kettering Cancer Center, New York, NY USA; 5 Marie-Josée and Henry R. Kravis Center for Molecular Oncology, Memorial Sloan Kettering Cancer Center, New York, NY USA; 6 Department of Medicine, Memorial Sloan Kettering Cancer Center, New York, NY USA; 7 Weill Cornell Medical Center, New York, NY USA; 8 Department of Radiology, Memorial Sloan Kettering Cancer Center, New York, NY USA; 9 Department of Information Systems, Memorial Sloan Kettering Cancer Center, New York, NY USA; 10 Immunology Program, Memorial Sloan Kettering Cancer Center, New York, NY USA; 11 Human Oncology and Pathogenesis Program, Memorial Sloan Kettering Cancer Center, New York, NY USA; 12 Department of Radiation Oncology, Memorial Sloan Kettering Cancer Center, New York, NY USA; 13 Present Address: Bristol Myers Squibb, Princeton, NJ USA

**Keywords:** Cancer microenvironment, Cancer genomics, Genomic instability

## Abstract

High-grade serous ovarian cancer (HGSOC) is an archetypal cancer of genomic instability^1–4^ patterned by distinct mutational processes^5,6^, tumour heterogeneity^7–9^ and intraperitoneal spread^7,8,10^. Immunotherapies have had limited efficacy in HGSOC^11–13^, highlighting an unmet need to assess how mutational processes and the anatomical sites of tumour foci determine the immunological states of the tumour microenvironment. Here we carried out an integrative analysis of whole-genome sequencing, single-cell RNA sequencing, digital histopathology and multiplexed immunofluorescence of 160 tumour sites from 42 treatment-naive patients with HGSOC. Homologous recombination-deficient HRD-Dup (*BRCA1* mutant-like) and HRD-Del (*BRCA2* mutant-like) tumours harboured inflammatory signalling and ongoing immunoediting, reflected in loss of HLA diversity and tumour infiltration with highly differentiated dysfunctional CD8^+^ T cells. By contrast, foldback-inversion-bearing tumours exhibited elevated immunosuppressive TGFβ signalling and immune exclusion, with predominantly naive/stem-like and memory T cells. Phenotypic state associations were specific to anatomical sites, highlighting compositional, topological and functional differences between adnexal tumours and distal peritoneal foci. Our findings implicate anatomical sites and mutational processes as determinants of evolutionary phenotypic divergence and immune resistance mechanisms in HGSOC. Our study provides a multi-omic cellular phenotype data substrate from which to develop and interpret future personalized immunotherapeutic approaches and early detection research.

## Abstract (web summary)

Multi-modal analysis of genomically unstable ovarian tumours characterizes the contribution of anatomical sites and mutational processes to evolutionary phenotypic divergence and immune resistance mechanisms.

## Main

The principal defining features of high-grade serous ovarian cancer (HGSOC) are profound structural variations in the form of copy number alterations and genomic rearrangements, which accrue on a genetic background of nearly ubiquitous *TP53* mutation^14^. Somatic and germline alterations in homologous recombination (HR) repair pathway genes such as *BRCA1* and *BRCA2* lead to HR deficiency (HRD) in approximately half of HGSOC cases^15^. Beyond gene alterations, patients stratify by endogenous mutational processes^3,16^ as inferred from structural variation patterns in whole-genome sequencing (WGS), including HRD subtypes (*BRCA1*-associated tandem duplications, HRD-Dup; *BRCA2*-associated interstitial deletions, HRD-Del), *CCNE1* amplification-associated foldback inversion (FBI)-bearing tumours and *CDK12*-associated tandem duplicator (TD)-bearing tumours^5,6^.

HGSOC presents a distinctive clinical challenge resulting from the widespread intraperitoneal disease at diagnosis. Long latency allows for broad periods of clonal diversification and tumour–immune interactions to unfold in the heterogeneous microenvironments of the peritoneal cavity^7,10,17^. This raises key questions about how underlying mutational processes and local tissue sites influence clonal selection, tumour microenvironments (TMEs) and immune recognition. We carried out a prospective study, capturing mutational processes from WGS, cell phenotypes from single-cell RNA sequencing (scRNA-seq) and spatial topology from in situ multiplexed cellular imaging in multi-site cases of HGSOC. Our findings identify distinct immunostimulatory and immunosuppressive mechanisms that co-segregate with sites of disease and mutational processes, thereby defining new determinants of immune recognition and escape in HGSOC.

**Fig. 1:**
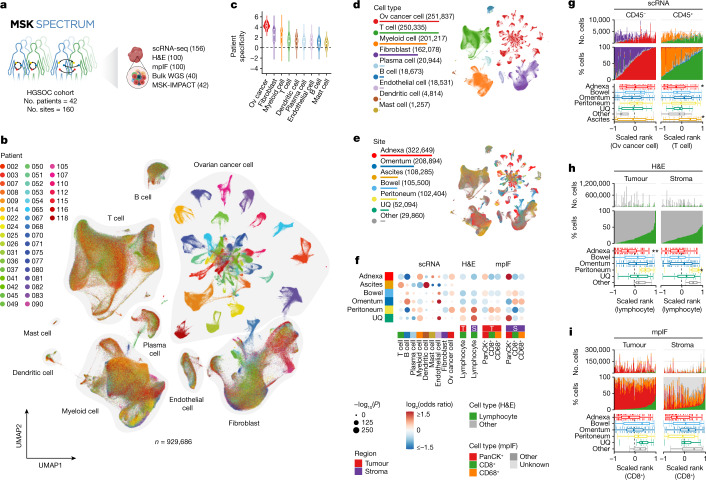
TME of HGSOC at single-cell resolution. **a**, Overview of the MSK SPECTRUM cohort and specimen collection workflow. **b**, UMAP plot of cells profiled by scRNA-seq coloured by patient. Cell types are highlighted with grey outlines. **c**, Patient specificity for each cell type (Methods). Ov, ovarian. **d**, Number of cells identified per cell type next to a UMAP plot with cells coloured by cell type. **e**, Number of cells profiled per tumour site next to a UMAP plot with cells coloured by tumour site. UQ, upper quadrant. **f**, Site-specific enrichment of cell type composition in scRNA-seq, H&E and mpIF data fitted using a GLM. GLMs for H&E and mpIF data were separated by tumour (T) and stroma (S) regions. The colour gradient indicates the log_2_-transformed odds ratio (red, enrichment; blue, depletion), and sizes indicate the Bonferroni-corrected –log_10_(*P* value). **g**, Cell type composition based on scRNA-seq data for CD45^−^ and CD45^+^ samples. Upper panels, absolute and relative cell type numbers; lower panels, box plot distributions of sample ranks with respect to tumour site. **h**, Cell type composition based on H&E with lymphocyte ranks in tumour and stroma. Panels are analogous to those in **g**. **i**, Cell type composition based on mpIF with CD8^+^ T cell ranks in tumour and stroma. Panels are analogous to those in **g**. For **c** and **g**–**i**, violin plots and box plots are shown as the median, top and bottom quartiles; whiskers correspond to 1.5× interquartile range (IQR). *P < 0.05, **P < 0.01.

**Extended Data Fig. 1:**
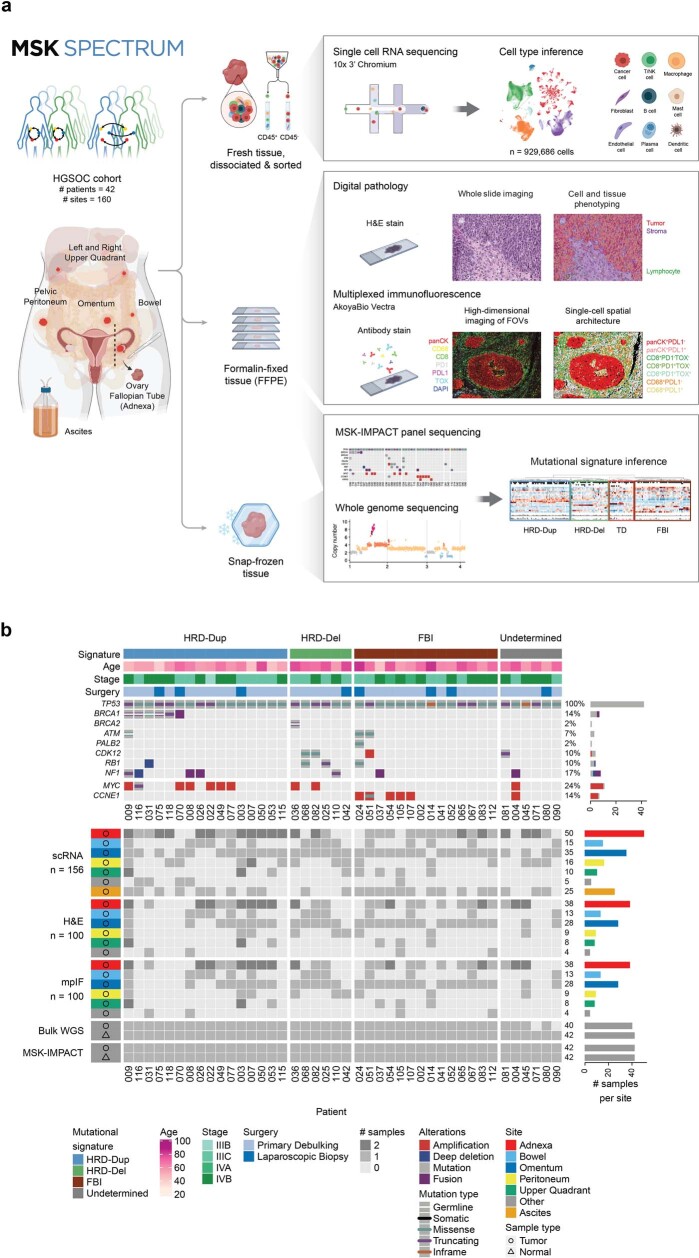
Multi-site, multi-modal profiling of malignant cells and the TME. **a**, Schematic of the MSK SPECTRUM specimen collection workflow including surgery, single-cell suspensions for scRNA-seq and biobanking of snap-frozen and FFPE samples. **b**, Cohort overview. Top panel, Oncoprint of selected somatic and germline mutations per patient and cohort-wide prevalence. Single nucleotide variants (SNVs), indels and fusions shown are detected by targeted panel sequencing (MSK-IMPACT). Focal amplifications and deletions are detected by whole-genome sequencing (WGS). Patient data include mutational signature subtype, patient age, staging following FIGO Ovarian Cancer Staging guidelines, and type of surgical procedure. Bottom panel, Sample and data inventory indicating number of co-registered multi-site datasets: single-cell RNA sequencing, H&E whole-slide images, multiplexed immunofluorescence, WGS and MSK-IMPACT.

**Extended Data Fig. 2:**
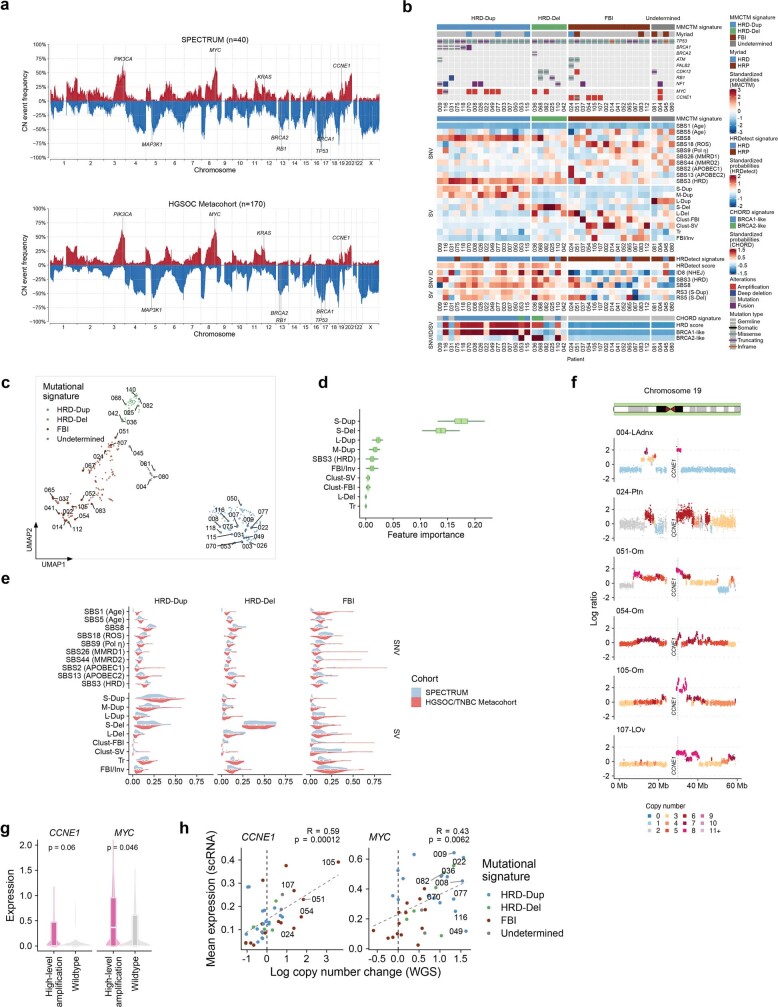
Inference of SNV and SV mutational signatures by WGS. **a**, Landscape of copy number gains and losses in the MSK SPECTRUM cohort (*n* = 40) and the HGSOC samples in the metacohort (*n* = 170). The fraction of tumour samples with gains or losses is shown on the *y*-axis, calculated in 500-kb genomic bins shown on the *x*-axis. **b**, First panel, Oncoprint of selected somatic and germline mutations per patient and cohort-wide prevalence by MSK-IMPACT. Patients are grouped by mutational signature. Second panel, heatmap of standardized probabilities for genomic features used to infer mutational signature subtypes from WGS using MMCTM. Features used for inference (in rows) are grouped into single nucleotide variant (SNV) and structural variation (SV) features. SV features include duplications (S-Dup, M-Dup, L-Dup), deletions (S-Del, L-Del), unclustered and clustered foldback inversions (FBI/Inv, Clust-FBI), clustered rearrangements (Clust-SV) and translocations (Tr). Third panel, standardized probabilities for SNV, indel and SV features used by HRDetect. Fourth panel, standardized probabilities for *BRCA1*- and *BRCA2*-like signatures used by CHORD. **c**, UMAP representation of SNV and SV features in the MSK SPECTRUM cohort (*n* = 40) and the HGSOC/TNBC metacohort (*n* = 309), coloured by signature strata. Patient identifiers of SPECTRUM cases are highlighted. **d**, Ranked SNV and SV feature importance in the classification of signature strata. Violin plots show permutation-based importance estimates over randomly shuffled signature strata. **e**, Paired violin plot of SNV and SV signature probabilities estimated by MMCTM, showing a comparison between the MSK SPECTRUM cohort and the HGSOC/TNBC metacohort. **f**, Chromosome 19 copy number shown using the log_2_ ratio (*y*-axis) for individual genomic bins (*x*-axis), coloured by the copy number state. A chromosome ideogram highlights the region of interest in chromosome 19. The *CCNE1* locus is marked with a dashed line. Patients are annotated to show whether *CCNE1* amplification was predicted by WGS. Only patients with a focal *CCNE1* amplification called by WGS were included. **g**, Violin plot of single-cell expression of oncogenes in scRNA-seq, stratified by oncogene copy number in site-matched WGS. **h**, Correlation between log_2_ CN change in oncogenes profiled by WGS and mean expression in cancer cells based on matched scRNA-seq from CD45^−^ samples. Spearman’s rank correlation coefficient for the linear fit is shown. Patients are coloured by mutational signature, and those highlighted have high-level amplifications detected by WGS. In **d**, **e** and **g**, box plots show the median, top and bottom quartiles; whiskers correspond to 1.5× IQR.

Multi-site tissue biopsies (*n* = 160) were collected from newly diagnosed, treatment-naive patients (*n* = 42) undergoing laparoscopy or primary debulking surgeries over a 24-month period (Fig. 1a). Collection took place at anatomical sites including adnexa (that is, potential primary lesions), omentum, peritoneum, bowel, ascites and other intraperitoneal sites (Extended Data Fig. 1a). The clinical characteristics of all patients are summarized in Extended Data Fig. 1b and Supplementary Table 1. Patient samples were profiled using scRNA-seq on CD45^+^ and CD45^−^ flow-sorted fractions (Supplementary Table 2), haematoxylin and eosin (H&E) staining and multiplexed immunofluorescence (mpIF) on fixed tissue sections, clinical tumour–normal sequencing of 468 cancer genes by MSK-IMPACT and whole-genome tumour–normal sequencing (Extended Data Fig. 1a,b and Methods). WGS copy number profiles were highly concordant with an external ‘meta-cohort’ derived from several HGSOC WGS studies^5^ (Extended Data Fig. 2a). Mutational signature inference from WGS data yielded 16 HRD-Dup, 6 HRD-Del and 14 FBI tumours (Extended Data Figs. 1b and 2b,c and Supplementary Tables 1 and 3), with model features consistent with previous reports^5^ (Extended Data Fig. 2d,e), stable across multiple computational methods^18,19^ and in agreement with *BRCA1* and *BRCA2* mutations and clinical HRD testing (Extended Data Fig. 2b). Furthermore, tumours with high-level amplifications in *CCNE1* (Extended Data Fig. 2f) and *MYC* exhibited expected distributions of gene amplifications within signature assignments and *cis*-acting gene expression correlates (Extended Data Fig. 2g,h).

## Site-specific TMEs

**Extended Data Fig. 3:**
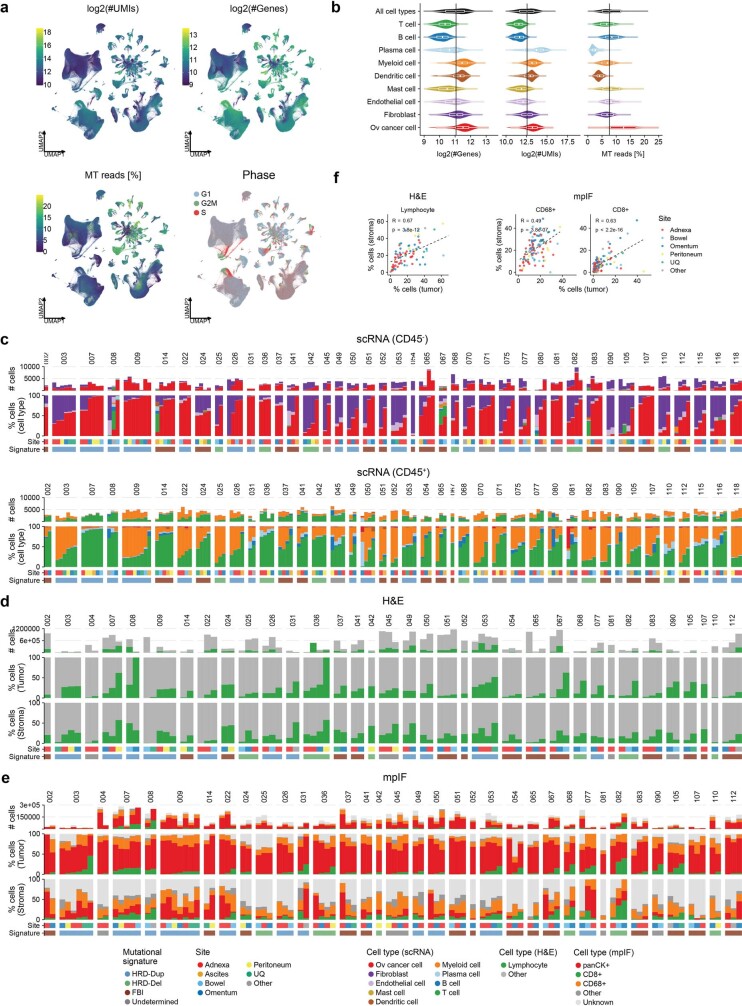
Quality control of scRNA-seq data and cell type abundance profiled by scRNA-seq, H&E and mpIF. **a**, UMAPs of cells profiled by scRNA-seq coloured by different QC metrics: log_2_ transformed number of UMIs and genes, fraction of mitochondrial reads, cell cycle phase. **b**, Distributions of QC metrics per cell type. Box plots show the median, top and bottom quartiles; whiskers correspond to 1.5× IQR. **c**, Absolute and relative cell type compositions of CD45^−^ (top) and CD45^+^ (bottom) sorted samples based on scRNA-seq, separated by patient, ranked by fraction of ovarian cancer cells and T cells respectively. **d**, Absolute and relative cell type compositions based on H&E, ranked by lymphocyte fractions for tumour-rich (top) and stroma-rich (bottom) compartments. Panel analogous to **c**. **e**, Absolute and relative cell type compositions based on mpIF, ranked by CD8^+^ T cell fractions in tumour-rich (top) and stroma-rich (bottom) compartments. Panel analogous to **c**. Colour legends for **c**–**e** are shown along the bottom of the figure. **f**, Correlation between the fraction of lymphocytes in tumour and stroma regions of H&E slides (left panel) and the fraction of CD8^+^ and CD68^+^ cells in mpIF slides (right panel).

**Extended Data Fig. 4:**
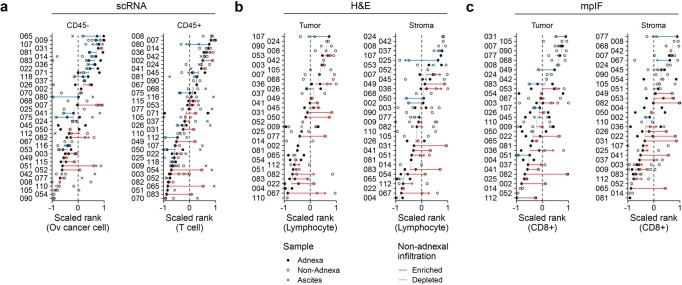
Intra-patient heterogeneity of HGSOC tumour microenvironments. **a**, Cell type composition based on scRNA-seq for CD45^−^ samples (left) and CD45^+^ samples (right). Dot plot of sample ranks grouped by patient. Coloured arrows indicate enrichment (red) or depletion (blue) of ovarian cancer cells (left) and T cells (right) in non-adnexal over adnexal samples. **b**, Cell type composition based on H&E with lymphocyte ranks in tumour-rich (left) and stroma-rich (right) compartments. Panels analogous to **a**. **c**, Cell type composition based on mpIF with CD8^+^ T cell ranks in tumour-rich (left) and stroma-rich (right) compartments. Panels analogous to **a**.

We constructed a cell type map from the scRNA-seq data, quantifying nine broad cellular lineages: epithelial cells, lymphoid cells (T and natural killer (NK) cells, B cells, plasma cells), myeloid cells (monocytes/macrophages, dendritic cells (DCs), mast cells) and stromal cells (fibroblasts, endothelial cells) (Fig. 1b,d, Extended Data Fig. 3a,b and Supplementary Table 4). Ovarian cancer cells exhibited high patient specificity (Fig. 1c and Methods), attributed to tumour cell-specific somatic copy number alterations driving gene dosage effects. Immune cell composition varied across anatomical sites within patients (Fig. 1e,f). Whereas CD45^−^ fractions (ranging from fibroblast-rich to cancer cell-rich samples) were largely conserved between anatomical sites (Fig. 1g), CD45^+^ fractions (ranging from myeloid-rich to lymphoid-rich samples) were substantially different (Fig. 1f,g and Extended Data Fig. 3c). Unsurprisingly, ascites samples were enriched for T cells (Mann–Whitney *U* test, Benjamini–Hochberg (BH)-corrected *Q* = 0.0195) and DCs (*Q* < 1 × 10^−4^), while adnexal samples were comparatively depleted for T cells (*Q* = 1.95 × 10^−2^), B cells (*Q* = 4.1 × 10^−3^) and DCs (*Q* = 6.0 × 10^−3^). Among solid tumour sites, higher lymphocyte and CD8^+^ T cell fractions were found in non-adnexal sites in scRNA-seq, whole-slide H&E and mpIF (Fig. 1g–i and Extended Data Figs. 3c–e and 4a–c) in both tumour and stromal regions (Extended Data Fig. 3f).

**Fig. 2:**
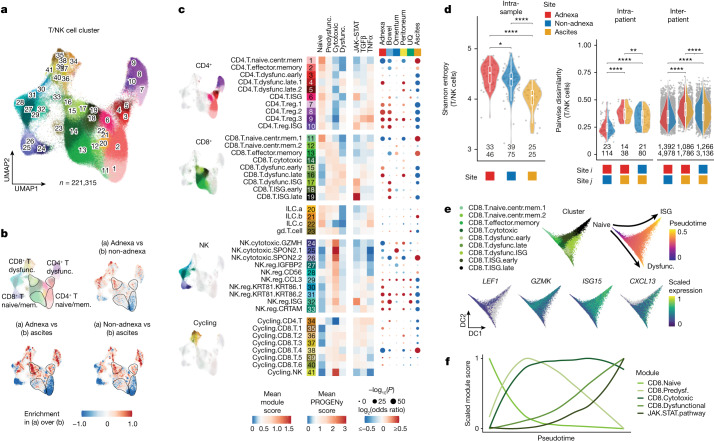
Site specificity of immunophenotypes. **a**, UMAP plot of T and NK cell clusters profiled by scRNA-seq. Clusters are coloured and numbered to reference cluster labels in **c**. **b**, Pairwise comparisons of kernel density estimates in UMAP space. **c**, Left, heatmap of average T cell state module scores (left) and signalling pathway activity scores (right) across CD4^+^ T, CD8^+^ T, innate lymphoid cell (ILC), NK and cycling cell clusters. Right, dot plot showing site-specific enrichment of T and NK cell clusters based on GLM. The colour gradient indicates the log_2_-transformed odds ratio (red, enrichment; blue, depletion), and sizes indicate the Bonferroni-corrected –log_10_(*P* value). **d**, Intra-sample diversity of T and NK cell clusters estimated by Shannon entropy with samples grouped by site (patient and sample counts shown) and intra- and inter-patient dissimilarity of T and NK cell cluster composition for pairs of samples, estimated using the Bray–Curtis distance (patient and sample pair counts shown). Pairwise dissimilarity is shown for all heterotypic pairs of sites (adnexa versus non-adnexa, adnexa versus ascites, non-adnexa versus ascites). Violin plots show the median, top and bottom quartiles; whiskers correspond to 1.5× IQR. *P < 0.05, **P < 0.01, ***P < 0.001, ****P < 0.0001. **e**, Top, diffusion maps of the subset of CD8^+^ T cells profiled by scRNA-seq, with cells coloured by CD8^+^ T cell cluster and pseudotime. Bottom, relative expression of genes marking CD8^+^ T cell clusters in diffusion space. DC, diffusion component. **f**, Scaled module scores with respect to pseudotime.

**Extended Data Fig. 5:**
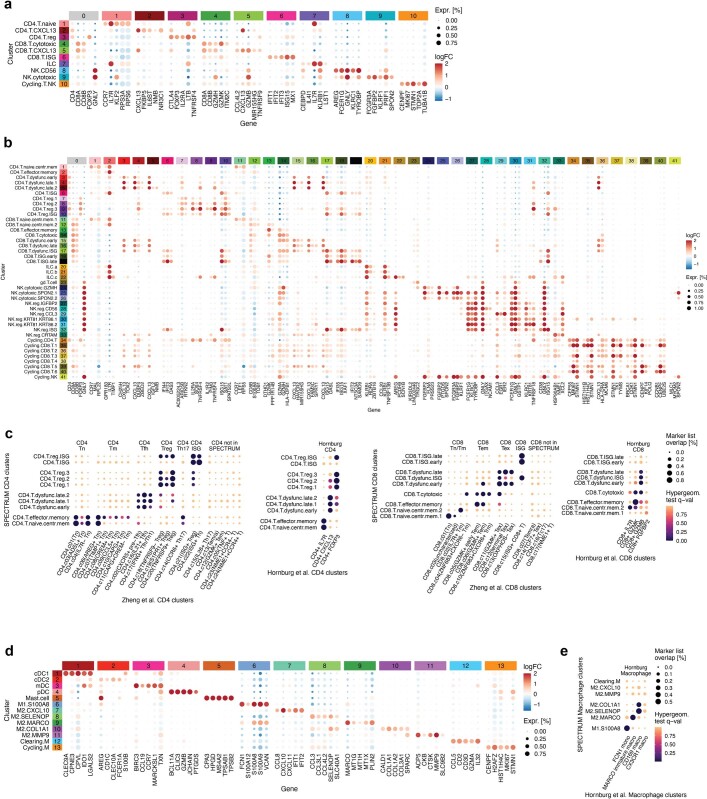
Marker gene expression of T, NK and myeloid cell phenotypes. **a**, Heatmap of scaled marker gene expression (averaged per cluster) for coarse-grained T and NK cell clusters, showing differentially expressed genes in columns and clusters in rows. Genes are grouped by cluster. Top 5 genes per cluster are highlighted. **b**, Panel analogous to **a**, for fine-grained T and NK cell clusters. Top 3 genes per cluster are highlighted. **c**, Comparison of SPECTRUM T cell clusters (this study) and published T cell clusters using hypergeometric test to assess statistical significance of cluster marker gene overlap. **d**, Marker gene expression heatmap for myeloid cells (dendritic cells, mast cells and macrophage clusters). Top 5 genes per cluster are highlighted. **e**, Comparison of SPECTRUM macrophage clusters (this study) and published macrophage clusters using hypergeometric test to assess statistical significance of cluster marker gene overlap.

Inter-site compositional variation within patients stimulated deeper analyses to assess immune cell phenotypic states. We identified 10 major T and NK cell clusters with 41 minor subclusters (Fig. 2a, Extended Data Fig. 5a,b and Supplementary Table 4), broadly defining CD4^+^ T cells (clusters 1–10), CD8^+^ T cells (clusters 11–19), innate-like and γδ T cells (clusters 20–23), NK cells (clusters 24–33) and cycling cells (clusters 34–41). Clusters were annotated on the basis of known marker genes and cross-referenced against other published annotations^20,21^ (Extended Data Fig. 5c). T and NK cell clusters followed a gradient across uniform manifold approximation and projection (UMAP) space (Fig. 2b), highlighting site-specific phenotypic differences that were quantified by fitting a generalized linear model (GLM) of cluster composition (Fig. 2c). In particular, naive/stem-like and central memory CD4^+^ T cells (cluster 1) were depleted in adnexal samples and enriched in ascites (Extended Data Fig. 7a,b and Supplementary Table 5). Conversely, dysfunctional CD4^+^ and CD8^+^ T cells (clusters 3–5 and 15–17) were depleted in ascites but enriched in adnexal and other tumour sites (Supplementary Table 5), in line with dysfunction driven by chronic antigen exposure in solid tumours. Clusters for regulatory T cells (7–10) and regulatory NK cells (27–33) were also enriched in adnexal samples (Fig. 2c), potentially indicative of increased immunomodulatory feedback at these sites.

Comparisons of solid tumour sites within patients showed naive/stem-like and central memory T cell enrichment in non-adnexal sites (22 of 31 patients) and dysfunctional T cell enrichment in adnexal sites (19 of 31 patients) (Extended Data Fig. 7a, vector plot). Shannon entropy analysis indicated higher within-site variation of T cell phenotypes in adnexal samples, suggesting the coexistence of differentiated states within the primary site relative to non-adnexal samples (Fig. 2d). Intriguingly, analysis of site-to-site Bray–Curtis dissimilarity showed high compositional differences between solid tumours and ascites both within and between patients (Fig. 2d and Extended Data Fig. 7c). Differentiation trajectories projected CD8^+^ T cells along two axes of (1) terminally dysfunctional and (2) interferon-stimulated gene (ISG) T cell states (Fig. 2e), defined by loss of naive T cell markers and acquisition of dysfunctional and cytotoxic traits (Fig. 2f). The trajectories were associated with loss of transcription factors expressed in naive and central memory T cells (*TCF1* and *LEF1*) and acquisition of type I interferon (IFN) (*ISG15*), cytotoxic function (*GZMK*) and T cell dysfunction (*TOX*, *CXCL13* and *PDCD1*) (Extended Data Fig. 7d). Notably, expression trajectories also differed across sites, with ascites exhibiting high cytotoxic module scores in contrast to the high dysfunctional T cell scores in adnexa and omentum (Extended Data Fig. 7e).

**Extended Data Fig. 6:**
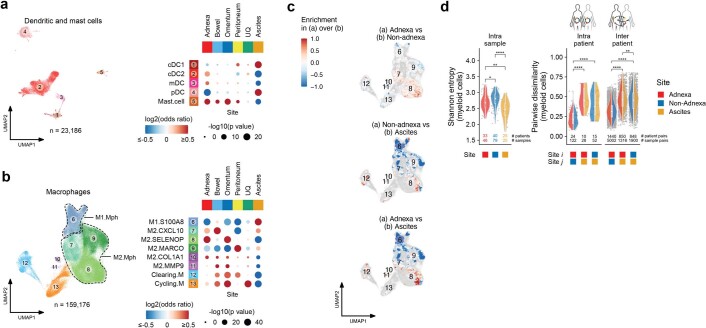
Anatomic site specificity of myeloid cell phenotypes. **a**, Left, UMAP of dendritic cells and mast cells. Clusters are coloured and numbered to reference cluster labels. Right, dot plot panel shows site-specific enrichment of DC and mast cell cluster composition using GLM. Colour gradient indicates log_2_ odds ratios (enrichment: red, depletion: blue) and sizes indicate the Bonferroni-corrected −log_10_(*P* value). **c**, Pairwise site differences in kernel density estimates in UMAP space for macrophages. **d**, Intra-sample diversity of myeloid cell clusters estimated by Shannon entropy grouped by site; and intra- and inter-patient dissimilarity of myeloid cell cluster composition between pairs of samples, estimated using the Bray-Curtis distance. Pairwise dissimilarity is shown between all heterotypic pairs of sites (i.e. adnexa/non-adnexa, adnexa/ascites, non-adnexa/ascites). Box plots show the median, top and bottom quartiles; whiskers correspond to 1.5× IQR. Brackets indicate two-sided Wilcoxon pairwise comparisons. *P < 0.05, **P < 0.01, ***P < 0.001, ****P < 0.0001.

**Extended Data Fig. 7:**
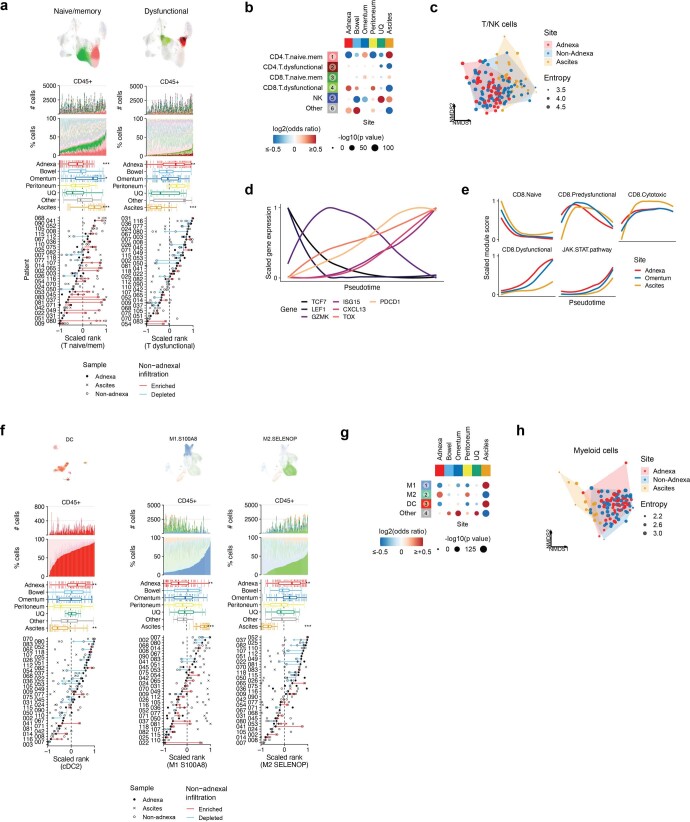
Intra-patient and inter-site heterogeneity of T, NK and myeloid cell phenotypes. **a**, T/NK cell cluster composition based on scRNA-seq, ranked by fraction of T naive/memory clusters (left) or fraction of T dysfunctional clusters (right). Panels analogous to Fig. 1g–i. **b**, Site-specific enrichment of coarse-grained T/NK cell clusters using GLM. Colour gradient indicates log_2_ odds ratios and sizes indicate the Bonferroni-corrected -log_10_(*P* value). **c**, Dimensionality reduction of the dissimilarity in T/NK cluster composition between pairs of samples using NMDS (**Methods**). Convex hulls highlight differences between samples based on the anatomic site. Size indicates the Shannon entropy in cluster composition per sample. **d**, Genes of interest in subsets of CD8^+^ T cells as a function of pseudotime inferred from diffusion components. **e**, Scaled module scores with respect to pseudotime, grouped by tumour site. **f**, Myeloid cell cluster composition. Ranked by fraction of cDC2 (left), M1.S1008 cells (middle) and M2.SELENOP cells (right). Panels analogous to Fig. 1g–i. **g**, Site-specific enrichment of coarse-grained myeloid cell clusters using GLM analogous to **b**. **h**, Dimensionality reduction of the dissimilarity in myeloid cluster composition between pairs of samples using NMDS (**Methods**). Convex hulls highlight differences between samples based on the anatomic site. Size indicates the Shannon entropy in cluster composition per sample.

Phenotypic state composition in myeloid and DC compartments also varied as a function of site (Extended Data Fig. 6a–c). DCs clustered into conventional DCs (cDC1s, cDC2s), mature cDCs (mDCs) and plasmacytoid DCs (pDCs), marked by expression of *CLEC9A*, *CLEC10A*, *BIRC3* and *PTGDS*, respectively (Extended Data Figs. 5d and 6a and Supplementary Table 4). In addition, six different clusters of classical and alternatively activated macrophages were identified^22,23^, as well as cycling macrophages (Cycling.M) and phagocytic macrophages (Clearing.M) (Extended Data Figs. 5d,e and 6b and Supplementary Table 4). Both GLMs and kernel density estimates of cluster composition highlighted inter-site differences (Extended Data Fig. 7f), including cDC2 and M2.SELENOP depletion in ascites and enrichment in adnexa (Extended Data Fig. 6a–c and Supplementary Table 5). Conversely, M1.S100A8 macrophage fractions were decreased in adnexa and increased in ascites (Extended Data Figs. 6b,c and 7f and Supplementary Table 5). Similarly to T cells, major compositional differences were noted between solid tumour foci and ascites both within and between patients (Extended Data Fig. 6d).

Thus, phenotypic immune state differentiation for both lymphoid and myeloid cells was strongly linked to tumour site, underlying both within- and between-patient variation in TMEs and providing clear evidence that ascites immunophenotypic composition is unrepresentative of solid tumours.

## Tumour cell phenotypic diversification

**Fig. 3:**
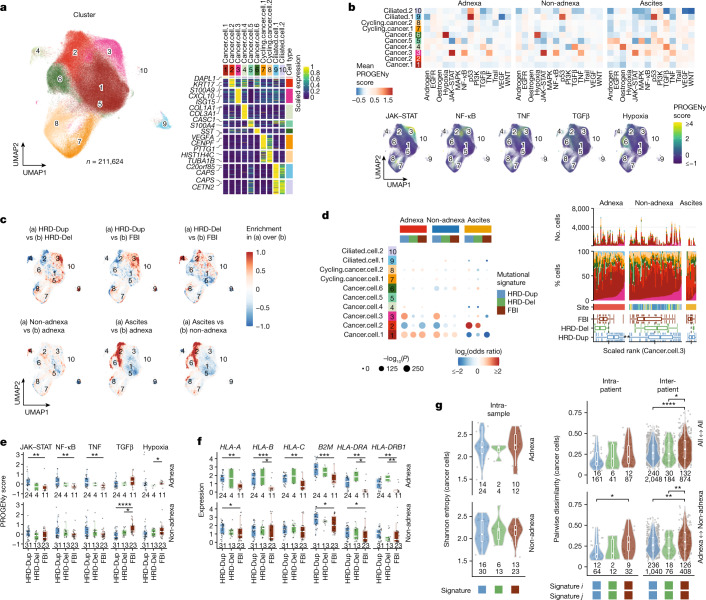
Malignant cell phenotypes and association with mutational signatures. **a**, Left, UMAP plot of epithelial cells coloured by cluster. Clusters are numbered to reference cluster labels in the heatmap. Right, heatmap of scaled marker gene expression averaged per cluster, showing differentially expressed genes in rows and clusters in columns. The top two genes for each cluster are highlighted. **b**, Top, heatmap of average signalling pathway activity scores per site. Bottom, UMAP plots with cells coloured by signalling activity scores for pathways of interest. EGFR, epidermal growth factor receptor; MAPK, mitogen-activated protein kinase; PI3K, phosphoinositide 3-kinase; VEGF, vascular endothelial growth factor. **c**, Relative kernel densities showing enrichment (red) and depletion (blue) in UMAP space for pairwise comparisons of mutational signatures and sites. **d**, Left, estimated effects of anatomical site and mutational signature on epithelial cluster composition based on GLM. The colour gradient indicates the log_2_-transformed odds ratio (red, enrichment; blue, depletion), and sizes indicate the Bonferroni-corrected –log_10_(*P* value). Right, epithelial cluster compositions ranked by Cancer.cell.3 fraction. Box plot panels show distributions of scaled sample ranks by mutational signature. **e**,**f**, Distributions of signalling pathway activity scores (**e**) and HLA gene expression (**f**) in adnexal and non-adnexal samples as a function of mutational signature (patient counts shown). **g**, Left, intra-sample diversity of malignant cell clusters in adnexal and non-adnexal samples, with samples grouped by mutational signature and site (patient and sample counts shown). Right, intra- and inter-patient dissimilarity of malignant cluster composition for pairs of samples. Pairwise dissimilarity is shown for all pairs of sites (patient and sample pair counts shown) excluding ascites (top) and for adnexal versus non-adnexal pairs of sites (bottom). In **d**–**g**, box plots and violin plots show the median, top and bottom quartiles; whiskers correspond to 1.5× IQR. Colours in **e**–**g** are analogous to those in **d**. *P < 0.05, **P < 0.01, ***P < 0.001, ****P < 0.0001; brackets indicate two-sided Wilcoxon pairwise comparisons in **e**–**g**.

**Extended Data Fig. 8:**
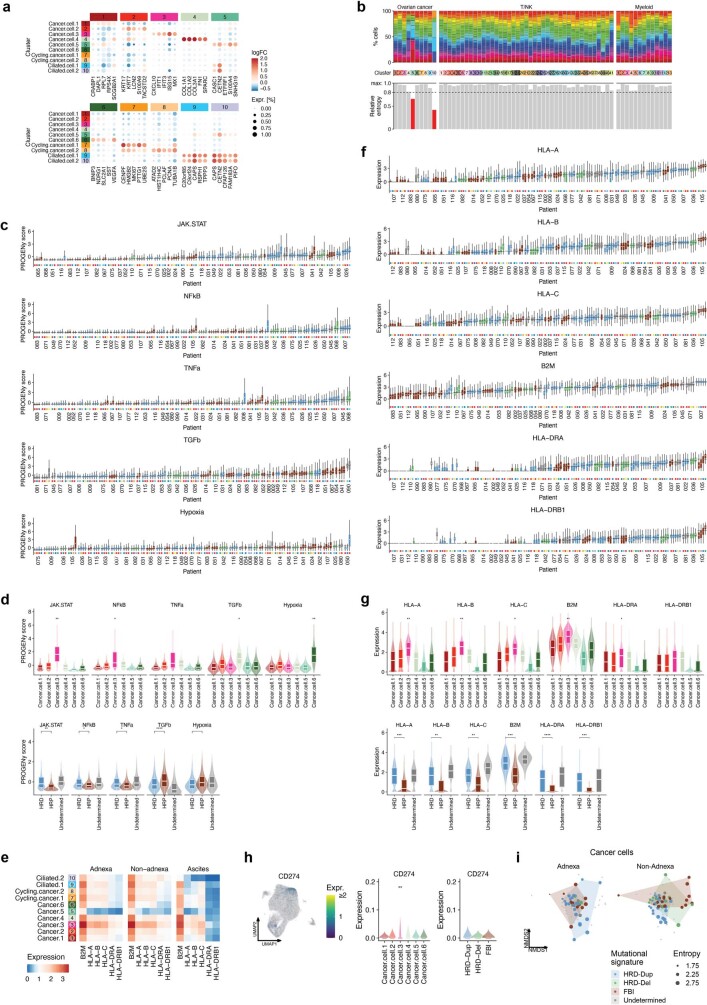
Mutational signatures impacting cancer cell-intrinsic signaling. **a**, Heatmap of scaled marker gene expression (averaged per cluster) for cancer cell clusters, showing differentially expressed genes in columns and clusters in rows. Genes are grouped by cluster. Top 5 genes per cluster are highlighted. **b**, Relative entropy of cell type subclusters to identify patient-specific clusters. **c**, Single cell distributions of PROGENy pathway activity per patient. **d**, Single-cell distributions of PROGENy pathway activity per cancer cell cluster (top subpanel), and as a function of HR status across all clusters (bottom subpanel). **e**, Heatmap of average HLA gene expression across clusters in adnexal, non-adnexal and ascites samples. **f**, Single cell distributions of HLA class II and class II gene expression per patient. **g**, Single cell distributions of HLA gene expression per cancer cell cluster (top subpanel), and as a function of HR status across all clusters (bottom subpanel). **h**, *CD274* (PD-L1) gene expression in UMAP space (left) and as box plot distributions (right) with respect to cluster and mutational signature respectively. **i**, Dimensionality reduction of the dissimilarity in cancer cell cluster composition between pairs of samples using NMDS. Convex hulls highlight differences between samples based on the anatomic site and mutational signature. Size indicates the Shannon entropy in cluster composition per sample. In **c**, **d** and **f**–**h**, box plots show the median, top and bottom quartiles; whiskers correspond to 1.5× IQR. Paired brackets in **d** and **g** show two-sided Wilcoxon pairwise tests. Group comparisons in **d**, **g** and **h** show one-sided Wilcoxon test. *P < 0.05, **P < 0.01, ***P < 0.001, ****P < 0.0001.

**Extended Data Fig. 9:**
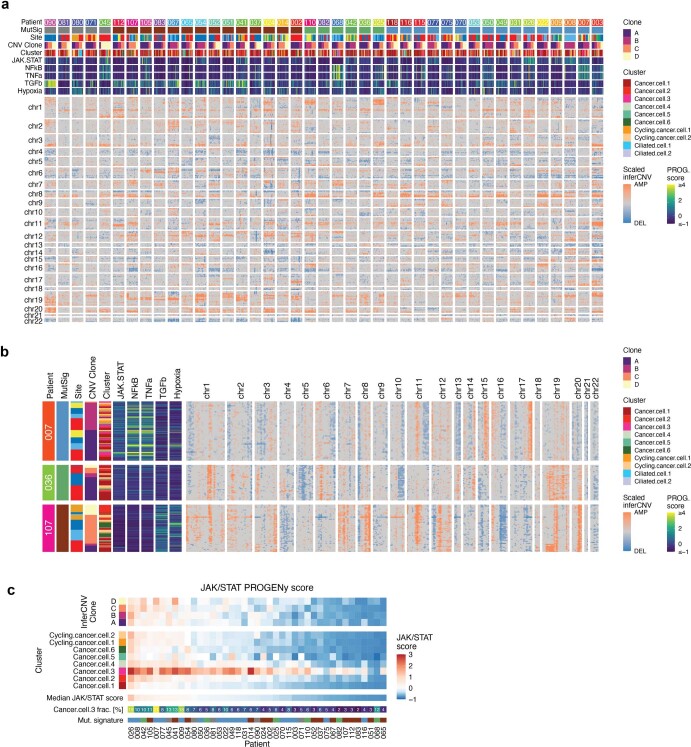
Clonal heterogeneity of chromosomal gains and losses in cancer cells. **a**, Copy number heatmap shows chromosomal gains and losses in cancer cells. Each column corresponds to an individual cell grouped by patient (*x*-axis), and chromosomes are arranged vertically (*y*-axis). Each patient’s dataset is downsampled to 50 cancer cells to facilitate visualization of the whole cohort. **b**, Examples of copy number heatmaps for all cancer cells from three patients. Rows correspond to individual cells grouped by patient (*y*-axis), and chromosomes are arranged horizontally (*x*-axis). **c**, Median JAK-STAT pathway activity in cancer cells, aggregated by patient (*x*-axis). From top to bottom (*y*-axis), median pathway scores are shown for cells grouped by inferCNV clone (top), transcriptional cancer cell cluster (middle) and median across all cancer cells (bottom).

We next defined how mutational processes in cancer cells influenced cancer cell-intrinsic signalling and immune phenotypes. We identified ten epithelialclusters from CD45^−^ cells (Fig. 3a, Extended Data Fig. 8a and Supplementary Table 4), including cells with elevated Janus kinase (JAK)–signal transducer and activator of transcription (STAT), nuclear factor (NF)-κB and tumour necrosis factor (TNF) signalling (Cancer.cell.3), transforming growth factor β (TGFβ) signalling (Cancer.cell.4) and hypoxia (Cancer.cell.6) (Fig. 3b). Mutational signature-specific cluster enrichments included Cancer.cell.3 in HRD-Dup and Cancer.cell.6 in FBI (Fig. 3c,d, Extended Data Fig. 8c,d and Supplementary Table 5). All three immune signalling pathways in Cancer.cell.3 were substantially increased in the adnexal lesions of HRD-Dup cases compared with FBI cases (Fig. 3e; *P* = 4.1 × 10^−3^, 5.2 × 10^−3^ and 5.2 × 10^−3^). This was not seen in non-adnexal lesions, implying that cell-intrinsic immune signalling in HRD-Dup cases originates in primary tumours. By contrast, TGFβ signalling was more prominent in non-adnexal sites of FBI cases (Fig. 3e; *P* < 1 × 10^−4^), linking FBI-specific activation of TGFβ signalling to the metastatic process. We note that within-patient differences in pathway activity were not linked to copy number clone identity (Extended Data Fig. 9a); for example, we see differences in JAK–STAT pathway activity independently of the copy-number profile in the same patient (Extended Data Fig. 9b,c).

Notably, cancer cell clusters differed by expression of major histocompatibility complex (MHC)-encoding genes (Fig. 3f and Extended Data Fig. 8e–g). MHC class I genes (*HLA-A*, *HLA-B*, *HLA-C* and *B2M*) and MHC class II genes (*HLA-DRA* and *HLA-DRB1*) were highly expressed in Cancer.cell.3, with upregulation in HRD relative to FBI adnexal tumours (Fig. 3f), indicative of increased antigen presentation accompanied by upregulated expression of *CD274* (PD-L1) (Extended Data Fig. 8h; *P* = 2.8 × 10^−3^). While at the sample level Shannon entropy showed similar levels of cell-intrinsic diversification across the mutational signatures, FBI tumours exhibited statistically higher Bray–Curtis dissimilarity in adnexa versus non-adnexa sample pairs (Fig. 3g and Extended Data Fig. 8i), potentially indicating that these cancer cells have a greater capacity for phenotypic diversity when migrating to distal sites.

**Fig. 4:**
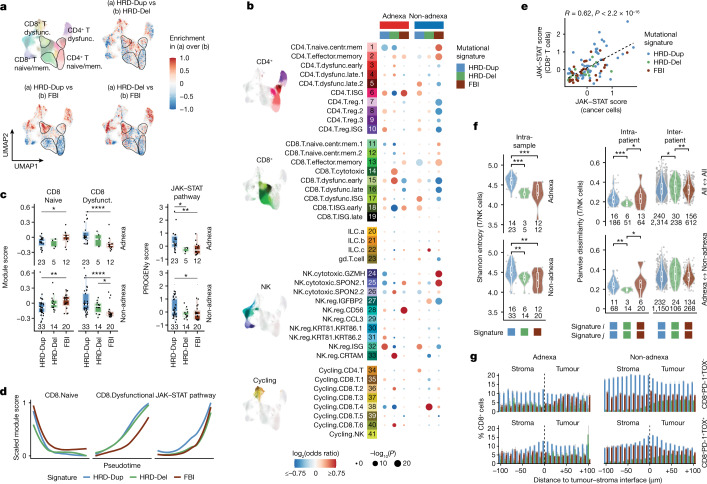
Mutational signatures as determinants of immunophenotypes. **a**, Differences in kernel density estimates in UMAP space for pairwise comparisons of mutational signatures. **b**, Estimated effects of mutational signature and anatomical site on T and NK cell cluster composition based on a GLM, with models fitted excluding ascites samples. The colour gradient indicates the log_2_-transformed odds ratio (red, enrichment; blue, depletion), and sizes indicate the Bonferroni-corrected –log_10_(*P* value). **c**, Distributions of CD8^+^ T cell state module scores and JAK–STAT signalling pathway activity scores with respect to mutational signature (patient counts shown). **d**, Scaled module scores within the subset of CD8^+^ T cells with respect to pseudotime and mutational signature. **e**, Correlation of JAK–STAT signalling scores in CD8^+^ T cells in CD45^+^ samples with those in cancer cells in matched CD45^−^ samples. **f**, Left, intra-sample diversity of T and NK cell clusters in adnexal and non-adnexal samples estimated by Shannon entropy, with samples grouped by mutational signature (patient and sample counts shown). Right, intra- and inter-patient dissimilarity in T and NK cell cluster composition, with samples grouped by mutational signature, estimated using the Bray–Curtis distance. Pairwise dissimilarity is shown for all pairs of sites (patient and sample pair counts shown) excluding ascites (top) and for adnexal versus non-adnexal pairs of sites (bottom). **g**, Spatial density of CD8^+^ T cell phenotypes in adnexal and non-adnexal mpIF samples as a function of distance to the tumour–stroma interface, with samples grouped by mutational signature (Methods). In **c** and **f**, box plots and violin plots show the median, top and bottom quartiles; whiskers correspond to 1.5× IQR. Colours in **f** and **g** are analogous to those in **c**–**e**. *P < 0.05, **P < 0.01, ***P < 0.001, ****P < 0.0001; brackets indicate two-sided Wilcoxon pairwise comparisons in **c** and **f**.

**Extended Data Fig. 10:**
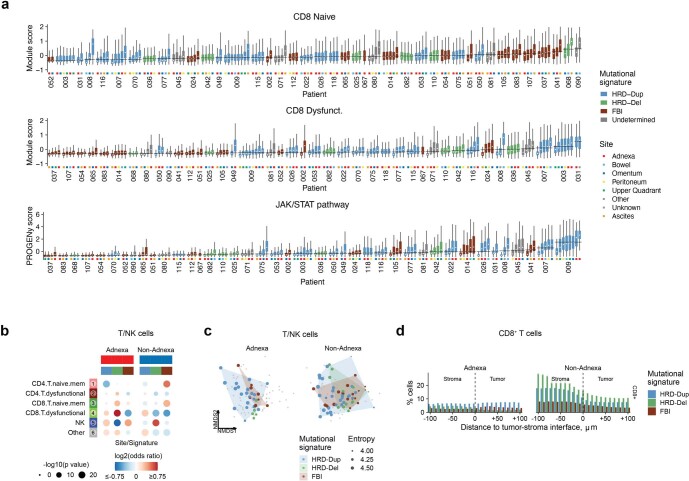
HR deficiency and tumour immunogenicity impact T cell phenotypes. **a**, Single cell distributions of T cell module scores per sample. Box plots are grouped by patient and coloured by mutational signature subtype. Samples are annotated based on the anatomic site of origin. **b**, Site and signature-specific enrichment of coarse-grained CD4^+^ T, CD8^+^ T and NK cell clusters using GLM. Colour gradient indicates log_2_ odds ratios and sizes indicate the Bonferroni-corrected −log_10_(*P* value). **c**, Dimensionality reduction of the dissimilarity in T/NK cluster composition between pairs of samples using NMDS (**Methods**). Convex hulls highlight differences between samples based on the anatomic site and mutational signature. **d**, Spatial density of CD8^+^ T cells in adnexal and non-adnexal samples as a function of distance to the tumour-stroma interface, grouped by mutational signature. Counts within 10 μm distance bands are grouped across FOVs from each mutational signature subtype, and are normalized by the total number of cells. Error bars denote the standard error of the proportion (**Methods**).

In addition, stark compositional differences of naive and dysfunctional T cells were observed as a function of mutational signature (Fig. 4a), with enrichment for naive/stem-like and central memory T cell clusters (1, 2, 11 and 12) in FBI tumours and dysfunctional T cells (3–5 and 15–17) in HRD tumours (Fig. 4b, Extended Data Fig. 10b and Supplementary Table 5). This was similarly reflected in higher JAK–STAT signalling in HRD-Dup tumours (Fig. 4c and Extended Data Fig. 10a) and along differentiation trajectories of T cell phenotypes (Fig. 4d). T cell-intrinsic and cancer cell-intrinsic JAK–STAT signalling was correlated in matched samples across the mutational subtypes (Fig. 4e). Higher phenotypic T cell state diversity was found in HRD-Dup tumours, accompanied by remarkably consistent intra-patient Bray–Curtis indices, suggesting that diversification processes were recurrent across patients (Fig. 4f).

Using mpIF, we tested whether heightened immune signalling in HRD tumours could be attributed to reciprocal interactions between cancer cells and immune cells in the tumour and stromal compartments in the TME (Fig. 4g and Extended Data Fig. 10d). Activated CD8^+^PD-1^+^TOX^−^ T cells were more prevalent in non-adnexal as compared with adnexal samples, with differences across compartments more pronounced in HRD subtypes than in FBI cases (Fig. 4g). Similarly, terminally dysfunctional CD8^+^PD-1^+^TOX^+^ T cells were enriched within the peritumoural stroma in HRD-Dup cases and in the tumour of HRD-Del cases. By contrast, CD8^+^PD-1^+^TOX^−^ and CD8^+^PD-1^+^TOX^+^ T cells were less abundant in FBI cases and were evenly distributed within the tumour and stroma, implying reduced T cell–antigen interactions (Fig. 4g).

**Extended Data Fig. 11:**
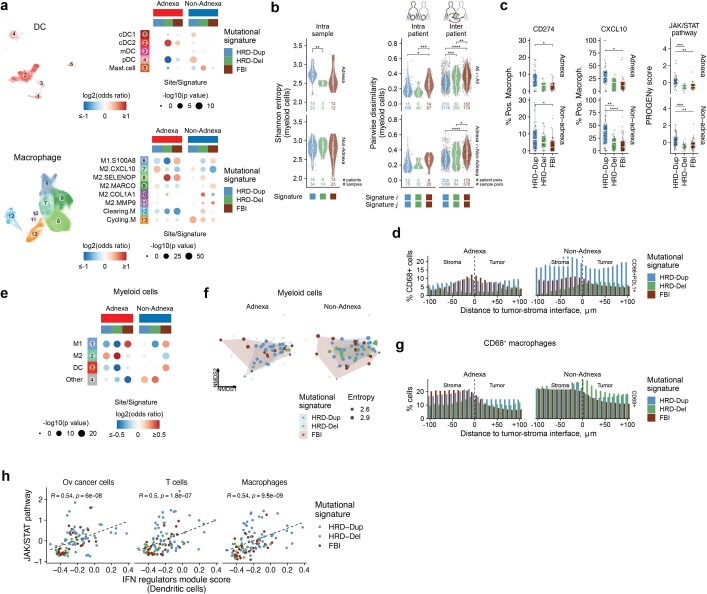
HR deficiency impacts myeloid cell phenotypes. **a**, Estimated effects of mutational signature on dendritic and macrophage cell cluster compositions using GLM. **b**, Left, intra-sample diversity of myeloid cell clusters in adnexal and non-adnexal samples estimated by Shannon entropy, with samples grouped by mutational signature (patient and sample counts shown). Right, intra- and inter-patient dissimilarity in myeloid cell cluster composition, with samples grouped by mutational signature, estimated using the Bray–Curtis distance. Pairwise dissimilarity is shown for all pairs of sites (patient and sample pair counts shown) excluding ascites (top) and for adnexal versus non-adnexal pairs of sites (bottom). **c**, Fraction of macrophages expressing *CD274* (PDL1) and *CXCL10* and JAK-STAT pathway activity with respect to mutational signature. **d**, Spatial density of CD68^+^PDL1^+^ macrophages in adnexal and non-adnexal mpIF samples as a function of distance to the tumour-stroma interface, grouped by mutational signature. Counts within 10 μm distance bands are grouped across FOVs from each mutational signature subtype, and are normalized by the total number of CD68^+^ cells. Error bars denote the standard error of the proportion (**Methods**). **e**, Site and signature-specific enrichment of coarse-grained myeloid cell clusters using GLM. Colour gradient indicates log_2_ odds ratios and sizes indicate the Bonferroni-corrected −log_10_(*P* value). **f**, Dimensionality reduction of the dissimilarity in myeloid cluster composition between pairs of samples using NMDS (**Methods**). Convex hulls highlight differences between samples based on the anatomic site and mutational signature. **g**, Spatial density of CD68^+^ macrophages in adnexal and non-adnexal samples as a function of distance to the tumour-stroma interface, grouped by mutational signature. Counts within 10 μm distance bands are grouped across FOVs from each mutational signature subtype, and are normalized by the total number of cells. Error bars denote the standard error of the proportion (**Methods**). **h**, Correlation between the fraction of dendritic cells expressing interferon regulating factors and JAK-STAT signaling scores in cancer cells, T cells and macrophages. In **b** and **c**, brackets indicate two-sided Wilcoxon pairwise comparisons. *P < 0.05, **P < 0.01, ***P < 0.001, ****P < 0.0001.

DC and macrophage phenotypic states were similarly shaped by tumour mutational signature, with cDC2 and M2.SELENOP enrichment in HRD-Del cases and enrichment of M1 macrophages in FBI cases (Extended Data Fig. 11a,e). Phenotypic diversification of myeloid cells was elevated in HRD-Dup adnexal samples with high entropy (Extended Data Fig. 11b, left); however, inter-patient Bray–Curtis dissimilarity was statistically higher in FBI tumours, suggesting greater patient specificity in FBI relative to HRD-Dup cases (Extended Data Fig. 11b, right). Diversification was characterized by M2.CXCL10 macrophage enrichment in HRD-Dup and depletion in FBI (Supplementary Table 5), with FBI tumours also exhibiting fewer PD-L1 (*CD274*)-positive macrophages (Extended Data Fig. 11a,d). In line with *CXCL10* being a target of JAK–STAT signalling, macrophages in HRD-Dup, but not FBI, samples presented higher JAK–STAT pathway activity (Extended Data Fig. 11c). Spatially, we observed elevated localization of CD68^+^ macrophages in the periphery of both HRD-Dup and FBI adnexal samples that extended into the tumour for HRD-Dup, but not FBI, samples (Extended Data Fig. 11g).

Concordance of JAK–STAT pathway activation among all cell subtypes implies a common upstream effector. We therefore examined type I IFN pathway regulators in DCs, which commonly serve as a key activator of JAK–STAT signalling. We observed a strong positive correlation between the IFN regulator module score in DCs and JAK–STAT pathway activation in cancer cells, T cells and macrophages (Extended Data Fig. 11h). Thus, increased type I IFN activation in DCs in HRD-Dup tumours may serve as an activator of JAK–STAT signalling, with downstream upregulation of human leukocyte antigen (HLA) molecules and PD-L1 in cancer cells and macrophages.

## Mutational processes drive immunoediting

**Fig. 5:**
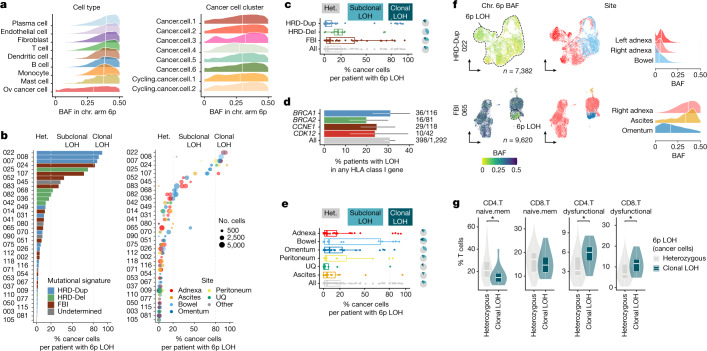
HLA loss as a mechanism of immune escape. **a**, Left, distribution over cells of chromosome arm 6p BAF in scRNA-seq data with ranking by median 6p BAF per cell type. Right, allelic imbalance in 6p BAF across cancer cell clusters. White vertical lines indicate the median. Chr., chromosome. **b**, Left, percentage of cancer cells with 6p LOH per patient. Right, site- and clone-specific percentage of cancer cells with 6p LOH. Het., heterozygous. **c**, Percentage of cancer cells with 6p LOH per sample as a function of mutational signature. Pie charts show the fraction of samples with heterozygous, subclonal LOH and clonal LOH 6p status. **d**, Percentage of patients with LOH of any HLA class I gene in the MSK-IMPACT HGSOC cohort (*n* = 1,298 patients) for *BRCA1*-, *BRCA2*- and *CDK12*-mutant and *CCNE1*-amplified tumours, mapping to HRD-Dup, HRD-Del, TD and FBI signatures, respectively. Error bars, 95% binomial confidence intervals. **e**, Percentage of cancer cells with 6p LOH per sample as a function of anatomical site. Pie charts show the fraction of samples by 6p status. **f**, UMAP plots of cancer cells from representative HRD-Dup and FBI cases. Density plots show site-specific 6p BAF. **g**, Fraction of naive and dysfunctional T cells in CD45^+^ samples as a function of the 6p LOH clonality of cancer cells in matched CD45^−^ samples. *P < 0.05; brackets indicate two-sided Wilcoxon pairwise comparisons. In **b**, **c**, **e** and **g**, 6p LOH status is defined as follows: heterozygous, percentage 6p LOH ≤ 20%; subclonal LOH, 20% < percentage 6p LOH ≤ 80%; clonal LOH, percentage 6p LOH > 80%. In **c**, **e** and **g**, box plots and violin plots show the median, top and bottom quartiles; whiskers correspond to 1.5× IQR. In **a**–**e**, only BAF estimates from cells with ≥10 reads aligning to 6p were considered and allelic imbalance states were assigned on the basis of the mean 6p BAF per cell as follows: balanced, BAF ≥ 0.35; imbalanced, 0.15 ≤ BAF < 0.35; LOH, BAF < 0.15 (Methods).

**Extended Data Fig. 12:**
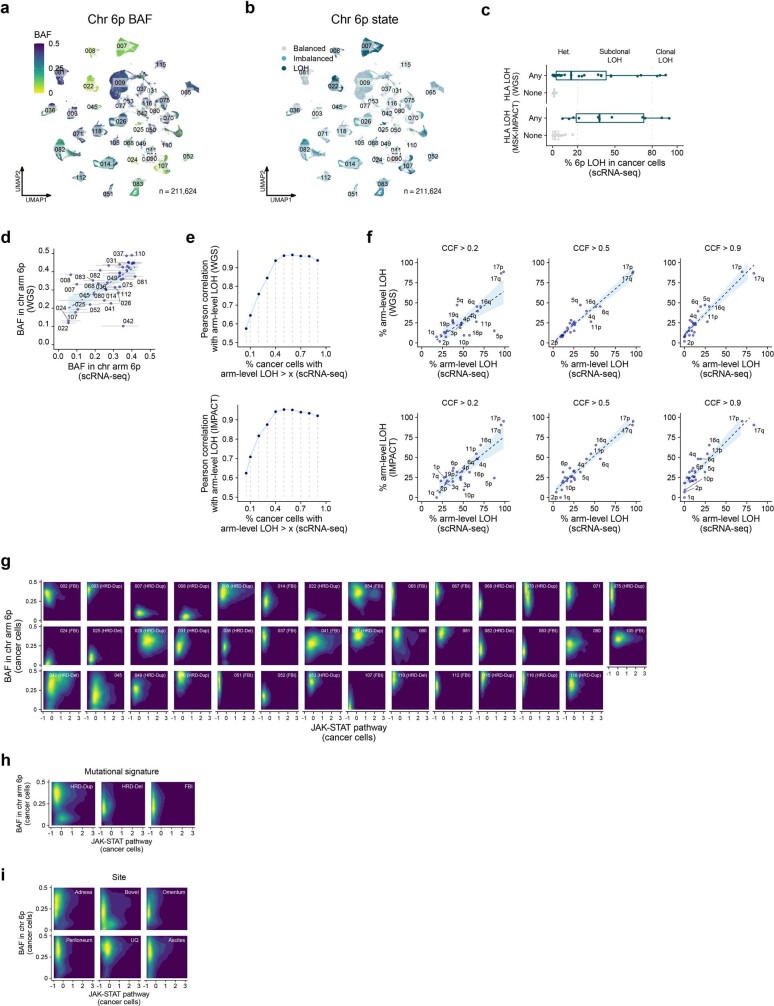
Intratumour heterogeneity of HLA loss of heterozygosity. **a**, UMAP of cancer cells profiled by scRNA-seq coloured by BAF of chromosome arm 6p. **b**, Allelic state of chromosome arm 6p. Allelic imbalance states per cell are assigned based on the mean 6p BAF per cell as balanced (BAF ≥ 0.35), imbalanced (0.15 ≤ BAF < 0.35) or LOH (BAF < 0.15) (**Methods**). **c**, Validation of 6p LOH estimates in cancer cells profiled by scRNA-seq using HLA LOH status in site-matched WGS samples (top) and site-matched MSK-IMPACT samples (bottom). 32 out of 41 patients profiled by scRNA-seq have site-matched WGS data, and 31 out of 41 patients have site-matched MSK-IMPACT data. Box plots show the median, top and bottom quartiles; whiskers correspond to 1.5× IQR. **d**, Correlation between arm-level BAF estimates inferred by scRNA-seq and WGS. **e**, Rarefaction curve of the arm-level LOH landscape detected by scRNA-seq, correlating the landscape of events at increasing cancer cell fractions in scRNA-seq to WGS (top) and MSK-IMPACT (bottom). **f**, Correlation between the fraction of samples with arm-level LOH in scRNA-seq and WGS (top), and in scRNA-seq and MSK-IMPACT (bottom), at increasing scRNA-seq cancer cell fractions. **g**, Normalized density contours of 6p BAF and JAK-STAT pathway activity in cancer cells for each patient. **h**–**i**, Normalized density contours of 6p BAF and JAK-STAT pathway activity in cancer cells comparing mutational signatures (**h**) and anatomic sites of origin (**i**). In **c**–**i**, only scRNA-seq BAF estimates from cells with ≥ 10 reads aligning to chromosome arm 6p are considered, and allelic imbalance states are assigned per cell based on the mean 6p BAF per cell as balanced (BAF ≥ 0.35), imbalanced (0.15 ≤ BAF < 0.35) or LOH (BAF < 0.15) (**Methods**).

We next investigated whether increased immune signalling in HRD subtypes led to mechanisms mediating immune escape. We profiled loss of HLA presentation machinery^24^ inferred through loss of heterozygosity (LOH) of chromosome arm 6p—harbouring HLA class I and class II genes—at the single-cell level using the SIGNALS algorithm^5^. Predictions were restricted to cancer cells (Fig. 5a), with per-cell B-allele fractions (BAFs) classed as balanced, imbalanced or LOH (Extended Data Fig. 12a,b) and orthogonal genomic validation from site-matched WGS and MSK-IMPACT datasets (Extended Data Fig. 12c–f). We observed marked inter-patient heterogeneity (Fig. 5b,c and Extended Data Fig. 12a,b), with clonal 6p LOH in 4 of 41 patients (10%) and subclonal 6p LOH in 7 of 41 patients (17%; Fig. 5c, left). Intriguingly, site-specific losses were found in 4 of 41 patients (Fig. 5c, right). Clonal 6p LOH was primarily observed in HRD-Dup cases, whereas subclonal distributions were more frequent in patients with FBI tumours (Fig. 5c). Higher prevalence of 6p LOH in HRD-Dup was validated in an independent cohort (*n* = 1,298 patients) with available MSK-IMPACT sequencing (31% in *BRCA1*-mutant cases, 19% in *BRCA2*-mutant cases and 24% in *CCNE1*-amplified cases Fig. 5d). Notably, clonal 6p LOH was present in adnexal lesions in 5 of 47 samples (Fig. 5e), in line with ‘early’ immune evolutionary selection in the primary site. Patient 022 with the HRD-Dup subtype and patient 065 with the FBI subtype further showed patient-specific evolutionary timing of 6p LOH (Fig. 5f). Functional consequences of 6p LOH in HRD-Dup were also observed, including upregulation of JAK–STAT signalling (Extended Data Fig. 12g,h), which was most pronounced in bowel samples (Extended Data Fig. 12i), and increased presence of dysfunctional CD4^+^ and CD8^+^ T cells (Fig. 5g). Together, association of LOH of HLA alleles with heightened JAK–STAT signalling and T cell dysfunction points to ‘early’ immune-mediated evolutionary selection of 6p loss in HRD-Dup tumours, in contrast to evolutionarily ‘late’ clonal expansion of 6p LOH in FBI tumours.

## Spatial topology of the microenvironment

**Extended Data Fig. 13:**
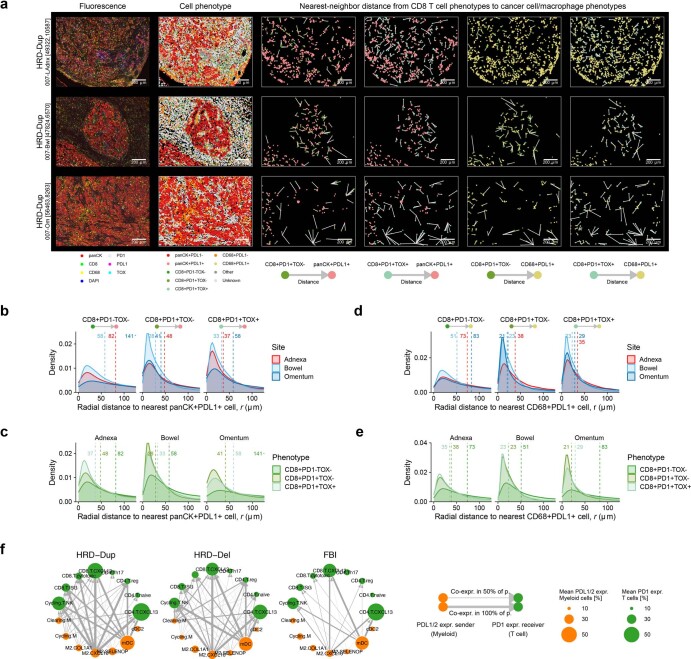
Site-specific determinants of spatial interactions between cancer cells, T cells and macrophages. **a**, Representative mpIF fields of view (FOVs) from adnexal, bowel and omentum specimens from patient 007 (HRD-Dup), indicating spatial intra-patient variation in ligand-receptor interactions between PD-L1 and PD-1. First column, raw pseudocolour images; second column, cellular phenotypes of segmented cells; remaining columns, proximity of pairs of phenotypes, highlighting ligand-receptor interactions between PD-L1 and PD-1 with colour-coded phenotypes, and edges depicting nearest-neighbour distances. Only edges joining pairs of cells within 250 μm are shown. **b**–**c**, Nearest-neighbour distance from CD8^+^ T cell phenotypes to panCK^+^PD-L1^+^ cancer cells aggregated across FOVs, grouped by anatomic site. Vertical lines indicate the median nearest-neighbour distance. **d**–**e**, Nearest-neighbour distance from CD8^+^ T cell phenotypes to CD68^+^PD-L1^+^ macrophages aggregated across FOVs, grouped by anatomic site. Vertical lines indicate the median nearest-neighbour distance. **f,** Interaction network diagrams depicting ligand-receptor co-expression across cell types, grouped by mutational signature. Nodes show mean PD-1 (*PDCD1*) expression in CD4^+^ T, CD8^+^ T and NK clusters, and mean PD-L1 (*CD274*) expression in myeloid cell clusters in scRNA-seq data, depicted by circle size. Arrows join ligand-expressing sender clusters to receptor-expressing receiver clusters and are weighted by frequency of PD-1 and PD-L1 co-expression.

The single-cell analyses above link immunophenotypic variation to mutational signatures and tumour site. We sought to validate these findings with tumour–immune cell interactions and spatial topologies from in situ mpIF profiling of principal immune cell types (T cells and macrophages) and their functional markers (PD-1, TOX, PD-L1) (Extended Data Fig. 13a). We enumerated the proximal interactions of naive/memory (CD8^+^PD-1^−^TOX^−^), activated/predysfunctional (CD8^+^PD-1^+^TOX^−^) and dysfunctional (CD8^+^PD-1^+^TOX^+^) T cells with PD-L1-expressing cancer cells (pan-cytokeratin (panCK)^+^PD-L1^+^). Interactions between proximal PD-L1-expressing cancer cells and activated/predysfunctional T cells were particularly high in bowel samples, and dysfunctional T cell interactions were high in both bowel and adnexal samples (Extended Data Fig. 13b,c). Omentum samples, by contrast, exhibited relatively few proximal interactions of either T cell or macrophage phenotypes with PD-L1-expressing cancer cells (Extended Data Fig. 13d,e).

**Fig. 6:**
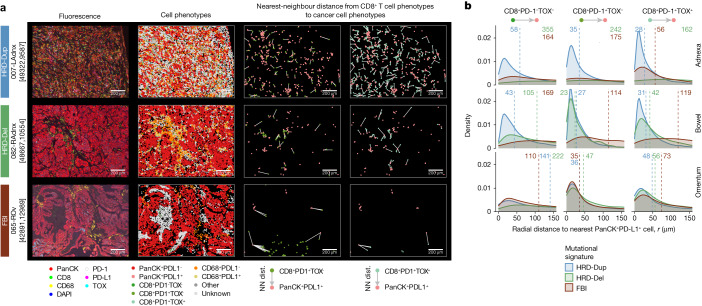
Spatial topologies of in situ cellular interactions. **a**, Representative mpIF fields of view (FOVs) highlighting common features of the TME and showing one adnexal sample per mutational signature. First column, raw pseudocolour images; second column, cellular phenotypes of segmented cells; third and fourth columns, proximity of phenotype pairs, highlighting PD-L1–PD-1 interactions with colour-coded phenotypes and edges depicting nearest-neighbour distances. Only edges joining pairs of cells within 250 μm of each other are shown. **b**, Nearest-neighbour distance from CD8^+^ T cell phenotypes to panCK^+^PD-L1^+^ cancer cells aggregated across FOVs, with samples grouped by anatomical site and mutational signature. Vertical lines indicate the median nearest-neighbour distance.

**Extended Data Fig. 14:**
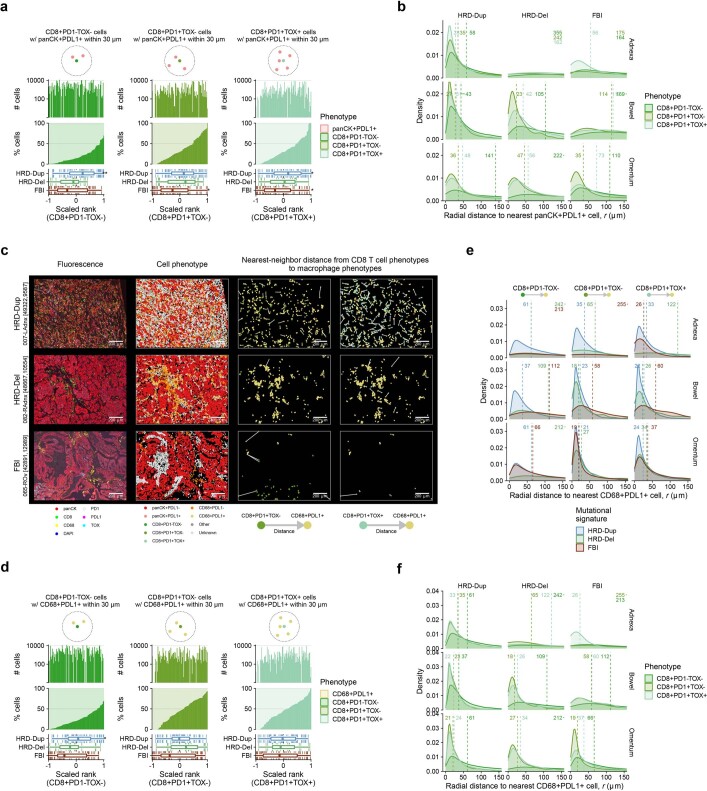
Mutational processes impacting spatial interactions between cancer cells, T cells and macrophages. **a**, Proximity analysis between CD8^+^ T cell phenotypes (green dots) and panCK^+^PD-L1^+^ cancer cells in their periphery (pink dots) based on mpIF data. Samples are ranked by the fraction of CD8^+^PD-1^−^TOX^−^ T cells (left), CD8^+^PD-1^+^TOX^−^ T cells (middle) or CD8^+^PD-1^+^TOX^+^ T cells (right) with ≥ 1 panCK^+^PD-L1^+^ cell within 30 μm. Upper panels, absolute abundance of CD8^+^ T cell states. Middle panels, fraction of CD8^+^ T cell phenotypes with ≥ 1 panCK^+^PD-L1^+^ cell within 30 μm. Lower panels, box plot distributions of sample ranks with respect to mutational signature. **b**, Nearest-neighbour distance from CD8^+^ T cell phenotypes to panCK^+^PD-L1^+^ cancer cells aggregated across fields of view (FOVs), grouped by anatomic site and mutational signature subtype. Vertical lines indicate the median nearest-neighbour distance. **c**, Representative mpIF FOVs highlighting common features of the tumour microenvironment, showing one adnexal sample per mutational signature subtype. First column: Raw pseudocolour images; second and third columns: proximity of pairs of phenotypes, highlighting ligand-receptor interactions between PD-L1 and PD-1 with colour-coded phenotypes, and edges depicting nearest-neighbour distances. Only edges joining pairs of cells within 250 μm are shown. **d**, Proximity analysis between CD8^+^ T cell phenotypes (green dots) and CD68^+^PD-L1^+^ macrophages (yellow dots) based on mpIF data, ranking samples by the fraction of CD8^+^PD-1^−^TOX^−^ T cells (left), CD8^+^PD-1^+^TOX^−^ T cells (middle) or fraction of CD8^+^PD-1^+^TOX^+^ T cells (right) with ≥ 1 CD68^+^PD-L1^+^ cell within 30 μm. Vertically aligned subpanels share the same *x*-axis. Upper panels, bar graphs show absolute abundance of CD8^+^ T cell states. Middle panels, bar graphs show the fraction of CD8^+^ T cell phenotypes with ≥ 1 CD68^+^PD-L1^+^ cell within 30 μm. Lower panels, box plot distributions of sample ranks with respect to mutational signature. **e**,**f**, Nearest-neighbour distance from CD8^+^ T cell phenotypes to CD68^+^PD-L1^+^ macrophages aggregated across FOVs, grouped by anatomic site and mutational signature subtype. Vertical lines indicate the median nearest-neighbour distance.

Mutational signatures also impacted cellular interactions, as predicted by higher receptor–ligand co-expression of PD-L1 (*CD274*) in myeloid clusters and PD-1 (*PDCD1*) in T and NK cell clusters in HRD subtypes derived from scRNA-seq data (Extended Data Fig. 13f). In line with these findings, the spatial organization of cellular neighbourhoods also varied by mutational subtype, as reflected in nearest-neighbour distances between T cells and panCK^+^PD-L1^+^ cancer cells (interactions of exemplar samples are shown in Fig. 6a). Antigen-experienced CD8^+^PD-1^+^ T cells within a 30-μm radius of PD-L1^+^ cancer cells were common in HRD-Dup cases but rare or absent in FBI tumours (Extended Data Fig. 14a). When combining site and signature, the shortest median distances were observed in HRD-Dup adnexa and bowel samples, particularly in the activated/predysfunctional and dysfunctional T cell compartments (Fig. 6b and Extended Data Fig. 14b), supporting PD-L1 as a negative feedback mechanism in response to activated T cells in HRD tumours. Similar interactions were noted between T cells and CD68^+^PD-L1^+^ macrophages, which were particularly prevalent in HRD-Del cases (Extended Data Fig. 14c–f), but largely absent in FBI tumours. Overall, mpIF analysis further highlighted site- and mutational signature-dependent TMEs consistent with scRNA-seq-based observations.

## Discussion

Our results synthesize anatomical sites and mutational processes as determinants of HGSOC TMEs and their phenotypic states. We speculate that, while the relative paucity of immune cells in adnexal sites is driven by the immune privilege of the ovaries and fallopian tubes, the predominance of dysfunctional T cells at these sites reflects immunoreactivity early in cancer evolution with subsequent immune escape in metastatic sites. In addition, contrasting cell-to-cell topological features of omental and bowel samples indicates that specific metastatic sites may harbour tissue-specific immunosurveillance constraints. Moreover, high intra- and inter-TME heterogeneity highlights that mechanisms of immune resistance are not universal in a given patient, requiring any therapeutic approach to account for evolution of the immune response in individual tumours.

Moreover, how mutational processes engender distinct immune evasion mechanisms raises additional questions in view of preclinical studies suggesting that immunogenicity in HRD tumours may lead to improved responses to immune checkpoint blockade (ICB)^25–28^. Clinical evidence for this is lacking as no association between HRD status, tumour mutational burden and response to ICB alone or in combination with chemotherapy has been observed^11–13^. Our findings highlight that mechanisms of immune resistance are distinct among the mutational subtypes, including activated type I IFN signalling in T cells, cancer cells and myeloid cells that is particularly enriched in HRD-Dup tumours. These data argue for multifaceted strategies for immunotherapeutic reprogramming that consider the underlying mutational process, in particular for FBI tumours, which are more resistant to chemotherapy^5^ and here are found to be immunologically inert.

Altogether, our study provides an extensive multi-modal resource, mapping the cellular constituents of HGSOC TMEs and linking them to mutational processes and spatial context. Our findings illustrate that even personalized approaches may be ineffective against widespread and heterogenous disease within patients, highlighting the urgent need for early detection before dissemination into the peritoneal cavity. The data presented here can be leveraged broadly to contextualize mechanistic insights into immunotherapeutic response across cancers of genomic instability.

## Methods

### Experimental methods

#### Sample collection

All enrolled patients were consented to an institutional biospecimen banking protocol and MSK-IMPACT^29^ testing, and all analyses were performed according to a biospecimen research protocol. All protocols were approved by the institutional review board (IRB) of Memorial Sloan Kettering Cancer Center. Patients were consented following the IRB-approved standard operating procedures for informed consent. Written informed consent was obtained from all patients before conducting any study-related procedures. The study was conducted in accordance with the Declaration of Helsinki and the Good Clinical Practice guidelines.

We collected fresh tumour tissues from 42 patients with HGSOC at the time of upfront diagnostic laparoscopic or debulking surgery. Ascites and tumour tissue from primary and multiple metastatic sites, including bilateral adnexa, omentum, pelvic peritoneum, bilateral upper quadrants and bowel, were procured in a predetermined, systematic fashion (median of four primary and metastatic tissues per patient) and were placed in cold RPMI for immediate processing. Blood samples were collected before surgery for the isolation of peripheral blood mononucleated cells (PBMCs) for normal WGS. The isolated cells were frozen and stored at −80 °C. In addition, tissue was snap frozen for bulk DNA extraction and tumour WGS. Tissue was also subjected to formalin fixation and paraffin embedding (FFPE) for histological, immunohistochemical and multiplex immunophenotypic characterization.

#### Sample processing

We profiled patient samples using five different experimental assays:

1. CD45^+^ and CD45^−^ flow-sorted cells were collected from fresh tissue samples and processed for scRNA-seq in 156 sites from 41 patients (~6,000 cells per site; Supplementary Table 2).
2. For each specimen with scRNA-seq data, site-matched FFPE tissue sections were used for whole-slide H&E staining and computational analysis (*n* = 100 tissue samples from 35 patients).
3. For each specimen with scRNA-seq data, site-matched FFPE tissue sections adjacent to the H&E section were stained by mpIF for major cell type and immunoregulatory markers (*n* = 1,349 quality-filtered FOVs across 100 tissue samples from 35 patients).
4. US Food and Drug Administration-approved clinical sequencing of 468 cancer genes (MSK-IMPACT) was performed on DNA extracted from FFPE tumour and matched normal blood specimens for each patient (Extended Data Fig. 1b).
5. Snap-frozen tissues were processed to obtain matched tumour–normal WGS data for a single representative site in *n* = 40 patients with scRNA-seq, H&E and mpIF data, to derive mutational processes from genome-wide single-nucleotide and structural variants.

#### scRNA-seq

##### Tissue dissociation

Tumour tissue was immediately processed for tissue dissociation. Fresh tissue was cut into 1-mm pieces and dissociated at 37 °C using the Human Tumor Dissociation kit (Miltenyi Biotec) on a gentleMACS Octo Dissociator. After dissociation, single-cell suspensions were filtered and washed with ammonium-chloride-potassium (ACK) lysing buffer. Cells were stained with trypan blue, and cell counts and viability were assessed using the Countess II Automated Cell Counter (ThermoFisher) (for a detailed protocol, see ref. ^30^).

##### Cell sorting

Freshly dissociated cells were stained with a mixture of GhostRed780 live/dead marker (TonBo Biosciences) and Human TruStain FcX Fc Receptor Blocking Solution (BioLegend). The stained samples were then incubated and stained with Alexa Fluor 700 anti-human CD45 antibody (BioLegend). After staining, cells were washed and resuspended in RPMI + 2% FCS and submitted for cell sorting. The cells were sorted into CD45^+^ and CD45^–^ fractions by fluorescence-assisted cell sorting on a BD FACSAria III flow cytometer (BD Biosciences). Positive and negative controls were prepared and used to set up compensations on the flow cytometer. Cells were sorted into tubes containing RPMI + 2% FCS for sequencing.

##### Library preparation

Flow-sorted tumour cells were stained with trypan blue, and the Countess II Automated Cell Counter (ThermoFisher) was used to assess both cell number and viability. Following quality control, the single-cell suspension was loaded onto a Chromium Chip B (10x Genomics, PN 2000060). GEM generation, cDNA synthesis, cDNA amplification and library preparation for 1,400–5,000 cells proceeded using Chromium Single-Cell 3′ Reagent kit v3 (10x Genomics, PN 1000075) according to the manufacturer’s protocol. cDNA amplification included 12 cycles, and 0.4–419 ng of the material was used to prepare sequencing libraries with 8–14 cycles of PCR.

##### Sequencing

Equimolar amounts of indexed libraries were pooled and sequenced on a HiSeq 2500 in rapid mode or on a NovaSeq 6000 in a 28-bp/91-bp, 100-bp/100-bp or 150-bp/150-bp paired-end run using HiSeq Rapid SBS kit v2 or NovaSeq 6000 SP, S1, S2 or S4 Reagent kit (100, 200 or 300 cycles) (Illumina).

#### Bulk WGS

##### Bulk tumour WGS

Frozen banked tissue was cut into sections on charged microscope slides. Following histological review, tumour tissue was microdissected if required to enrich for neoplastic cells^31^ and subjected to DNA extraction for bulk WGS. Genomic DNA was extracted using DNeasy Blood & Tissue kits (Qiagen) and quantified on a Qubit 3 Fluorometer using the Qubit 1× dsDNA HS Assay kit (Invitrogen).

##### Bulk normal WGS

PBMCs were brought up to a volume of 15 ml in cold PBS, and DNA was isolated with the DNeasy Blood & Tissue kit (Qiagen, 69504) according to the manufacturer’s protocol with 1 h of incubation at 55 °C for digestion. DNA was eluted in 0.5× buffer AE.

##### Sequencing

DNA quantity was measured using the Quant-iT PicoGreen dsDNA assay (ThermoFisher, P11496), and DNA quality was assessed with TapeStation D1000 ScreenTape (Agilent, 5067-5582). After PicoGreen quantification and quality control with an Agilent BioAnalyzer, 500 ng of genomic DNA was sheared using an LE220-plus focused ultrasonicator (Covaris, 500569) and sequencing libraries were prepared using the KAPA Hyper Prep kit (Kapa Biosystems, KK8504) with modifications. In brief, libraries were subjected to a 0.5× size selection using AMPure XP beads (Beckman Coulter, A63882) after postligation clean-up. Libraries were not amplified by PCR and were pooled in an equal volume and quantified on the basis of their initial sequencing performance. Samples were run on a NovaSeq 6000 in a 150-bp/150-bp paired-end run, using the NovaSeq 6000 SBS v1 kit and an S1, S2 or S4 flow cell (Illumina).

#### Preparation, review and scanning of histopathology slides

Archived FFPE tissues were used for histological review, including the assessment of spatial topology and tumour-infiltrating lymphocytes (TILs), as well as for immunohistochemical characterization and mpIF analysis for mapping of the TME, in the Advanced Immunomorphology Platforms Laboratory. Slides were originally reviewed by gynaecological pathologists for diagnosis and FIGO (International Federation of Gynecology and Obstetrics) stage assignment. Representative H&E-stained slides from each site of interest were digitally scanned to produce virtual slides. Two senior gynaecological pathologists then reviewed these images for the presence and location of serous tubal intraepithelial carcinoma (STIC), SET architecture (solid, pseudo-endometrioid and transitional cell-like patterns), micropapillary architecture^32^, presence of a fimbrial ball, architectural patterns of metastatic disease^33^, mitotic counts (per ten high-power fields, HPFs) and tumour cell content (viable percentage). Regions with TILs were also assessed with a quantitative TIL score (low, <42 TILs per HPF in a hotspot; high, 42 or more TILs per HPF in a hotspot)^32^. Histopathology slides were scanned into whole-slide images using a Leica Aperio AT2 scanner (Leica Biosystems) at ×20 magnification. The most representative tissue block was selected for slide scanning.

#### mpIF

##### Overview

We carried out multiparameter quantification of epithelial and immune cell subsets and activation markers using the AkoyaBio Vectra automated imaging system at the MSKCC Parker Institute for Cancer Immunotherapy. We stained whole slides of FFPE tissue for markers of ovarian cancer cells (panCK + CK8–CK18) and of specific leukocyte subsets, including macrophages (CD68) and cytotoxic T cells (CD8), known immune inhibitory proteins (PD-L1) and markers of the activation/exhaustion status of CD8^+^ T cells (PD-1, TOX). FOVs were chosen to include either the entire tissue with minimal field overlap if the tissue was small or a distribution of fields with 50% stroma/tumour at the edge plus some central areas of tumour-dense fields. Quality control was performed on marker intensities so that they fell in the range of 5–30 arbitrary units and helped guide spectral unmixing. Lower values might be close to background, while higher values prompted us to check for channel spillage.

##### Tissue staining

Primary antibody staining conditions were optimized using standard immunohistochemical staining on the Leica Bond RX automated research stainer with DAB detection (Leica Bond Polymer Refine Detection, DS9800). Using 4-µm FFPE tissue sections and serial antibody titrations, the optimal antibody concentration was determined followed by transition to a seven-colour multiplex assay with equivalency. Optimal primary antibody stripping conditions between rounds in the seven-colour assay were performed following a cycle of tyramide deposition followed by heat-induced stripping (see below) and subsequent chromogenic development (Leica Bond Polymer Regine Detection, DS9800) with visual inspection for chromogenic product with a light microscope by a senior pathologist. Multiplex assay antibodies and conditions are described in Supplementary Table 6.

Tissue sections were baked for 3 h at 62 °C in vertical slide orientation with subsequent deparaffinization performed on the Leica Bond RX followed by 30 min of antigen retrieval with Leica Bond ER2 and six sequential cycles of staining with each round including a 30-min combined block and primary antibody incubation (Akoya Antibody Diluent/Block, ARD1001).

For panCK and CK8–CK18, detection was performed using a secondary horseradish peroxidase (HRP)-conjugated polymer (Akoya Opal Polymer HRP Ms + Rb, ARH1001; 10-min incubation). Detection of all other primary antibodies was performed using a goat anti-mouse Poly HRP secondary antibody or goat anti-rabbit Poly HRP secondary antibody (Invitrogen, B40961 and B40962; 10-min incubation). The HRP-conjugated secondary antibody polymer was detected by fluorescent tyramide signal amplification using Opal dyes 520, 540, 570, 620, 650 and 690 (Akoya, FP1487001KT, FP1494001KT, FP1488001KT, FP1495001KT, FP1496001KT, FP1497001KT). The covalent tyramide reaction was followed by heat-induced stripping of the primary antibody–secondary antibody complex using PerkinElmer AR9 buffer (AR900250ML) and Leica Bond ER2 (90% ER2 and 10% AR9) at 100 °C for 20 min before the next cycle (one cycle of stripping for CD68, PD-1, PD-L1, CD8 and panCK/CK8/CK18 and two cycles of stripping for TOX). After six sequential rounds of staining, sections were stained with Hoechst (Invitrogen, 33342) to visualize nuclei and mounted with ProLong Gold antifade reagent mounting medium (Invitrogen, P36930).

##### Imaging and spectral unmixing

Seven-colour multiplex-stained slides were imaged using Vectra Multispectral Imaging System version 3 (PerkinElmer). Scanning was performed at ×20 magnification (×200 final magnification). Filter cubes used for multispectral imaging were DAPI, FITC, Cy3, Texas Red and Cy5. A spectral library containing the emitted spectral peaks of the fluorophores in this study was created using Vectra image analysis software (PerkinElmer). Using multispectral images from slides singly stained for each marker, the spectral library was used to separate each multispectral cube into individual components (spectral unmixing), allowing for identification of the seven marker channels of interest, using InForm 2.4 image analysis software.

### Computational methods

#### scRNA-seq

##### Overview

The pipeline was built using the 10x Genomics Martian language and computational pipeline framework. CellRanger software (version 3.1.0) was used to perform read alignment, barcode filtering and unique molecular identifier (UMI) quantification using the 10x GRCh38 transcriptome (version 3.0.0) for FASTQ inputs.

##### Quality control

CellRanger-filtered matrices were loaded into individual Seurat objects using the Seurat R package (version 3.0.1)^34,35^. The resulting gene-by-cell matrix was normalized and scaled for each sample. Cells retained for analysis had a minimum of 500 expressed genes and 1,000 UMI counts and had less than 25% mitochondrial gene expression. Cell cycle phase was assigned using the Seurat CellCycleScoring function. Scrublet (version 0.2.1) was used to calculate and filter cells with a doublet score greater than 0.25. Sample matrices were merged by patient and subsequently renormalized and scaled using default Seurat functions.

##### Major cell type identification

Major cell type assignments were computed for each patient with CellAssign (version 0.99.2)^36^ using a set of curated marker genes. Marker genes were compiled for nine major cell types related to HGSOC (Supplementary Table 4). These major cell types were defined as T cells, B cells, plasma cells, myeloid cells, DCs, mast cells, endothelial cells, fibroblasts and ovarian cancer cells. Before running CellAssign, cells with zero expression for all marker genes were removed from the count matrix. Cell-specific size factors were computed using scran (version 3.11). Default CellAssign parameters were used with a design matrix of patient batch labels. CellAssign returned a probability distribution over the major cell types, and individual cells were labelled by the resulting most probable cell type.

##### Dimensionality reduction

Principal-component analysis (PCA) was performed on the filtered feature-by-barcode matrix. UMAP embeddings including cohort-level and patient-level embeddings for all major cell types were based on the first 50 principal components. UMAP embeddings of major cell type supersets (see below) were based on the 50 batch-corrected harmony components. Diffusion map embeddings and pseudotime estimates were computed using the R package destiny (v3.0.1) for the subset of CD8^+^ T cells^37^.

##### Batch correction and integration

Major cell types identified across samples were split into six supersets: (1) T cells; (2) B cells and plasma cells; (3) myeloid cells, DCs and mast cells; (4) fibroblasts; (5) endothelial cells; and (6) ovarian cancer cells. For each superset, the R package harmony (version 0.1) was used for batch correction to account for patient-specific effects^38^.

##### Clustering and cell subtype identification

Graph-based clustering was performed for each superset using the Louvain algorithm implemented in Seurat (version 3.0.1) at three different resolutions (0.1, 0.2 and 0.3). Differential expression between identified clusters was computed using a two-sided Wilcoxon rank-sum test as implemented in Seurat FindMarkers. Final results were filtered on log(fold change) > 0.25 and Benjamini–Hochberg-adjusted *P* < 0.05. Clusters were annotated on the basis of marker genes identified in differential gene expression analysis. Patient-specific clusters not represented across the full cohort were identified using relative entropy. Relative entropy per cluster was defined as the maximum entropy per cluster divided by the empirical entropy of patient compositions. Clusters with a relative entropy of <0.8 were considered patient-specific clusters and disregarded for downstream analyses.

For T cell clusters, T cells and NK cells were clustered in two steps. Initial coarse-grained clustering resulted in ten different T and NK cell clusters, including four CD4^+^ T cell clusters, three CD8^+^ T cell clusters, two NK cell clusters and one cycling T/NK cell cluster (Extended Data Fig. 5a). Subclustering identified a total of 41 distinct fine-grained clusters, broadly defining major T cell and NK cell subtypes (Fig. 2a and Extended Data Fig. 5b). These included populations of CD4^+^ naive and central memory cells (expressing *IL7R* and *TCF7*), CD4^+^ effector memory cells (*IL7R*, *CCL5* and *KLRB1*), early and late dysfunctional CD4^+^ T cells (expressing dysfunctional T cell markers *CXCL13*, *TOX2* and *PDCD1*), regulatory T cells (*FOXP3* and *IL2RA*) and type 17 helper T cells (*KLRB1*, *RORA* and *RORC*). In the CD8^+^ compartment, we also identified populations of naive/central memory (expressing *KLF2*, *KLF3* and *TCF7*), activated/cytotoxic (*GZMH*, *GZMK* and *HLA-DR*) and early and late dysfunctional (*CXCL13*, *TOX2*, *LAG3*, *HAVCR2*, *TIGIT* and *PDCD1*) T cells. Notably, the early dysfunctional cluster, in addition to exhaustion-associated genes, was characterized by expression of *CXCR6* and *ITGAE*, commonly used to define tissue-resident memory T cells. In the innate compartment, we similarly identified several clusters, including a γδ T cell cluster and several NK cell clusters. Finally, in all compartments, we identified populations of cells marked by expression of type I IFN response genes such as *ISG15* and *IFIT3*, herein named CD4-ISG, CD8-ISG and NK-ISG, with strong upregulation of the JAK–STAT signalling pathway as the dominant feature of these cells (Fig. 2b). The remaining clusters consisted of cycling T and NK cells expressing S phase markers such as *MKI67* and G2M markers such as *TOP2A* (Supplementary Table 4).

For myeloid cell clusters, cDCs of the myeloid lineage were separated into cDC1s, cDC2s and mDCs, marked by expression of *CLEC9A*, *S100B* and *BIRC3*, respectively (Extended Data Figs. 5d and 6a). pDCs were marked by expression of *PTGDS*. Macrophage clusters were described with respect to their classical (M1-like) or alternative (M2-like) polarization. Six different clusters encompassing both classical and alternatively activated macrophages were identified, as well as a cluster of cycling macrophages (Cycling.M) and a cluster of actively phagocytic macrophages (Clearing.M). The M1-like and M2-like clusters were labelled according to the top genes defining the clusters (M1.S100A8, M2.CXCL10, M2.SELENOP, M2.MARCO, M2.COL1A1, M2.MMP9) (Extended Data Figs. 5d and 6b). Among these, the M1.S100A8 cluster was the only unambiguous M1-type macrophage cluster, marked by expression of pro-inflammatory calcium-binding protein genes *S100A8* and *S100A9*^22^. The M2.CXCL10 cluster was characterized by expression of both M1 (for example, *CXCL10*) and M2 (for example, *PDL1* and *C1QC*) markers. *CXCL10* is an established downstream target of type I and type II IFN signalling and was found to be expressed along with other CXC-motif chemokines (*CXCL9* and *CXCL11*). The remaining M2 clusters were all marked by high expression of complement component *C1QC*, which is known to promote M2 polarization^23^.

##### InferCNV copy number clonal decomposition

InferCNV (version 1.3.5)^39,40^ was used to identify large-scale copy number alterations in ovarian cancer cells classified by CellAssign. To do this, 3,200 non-cancer cells were randomly sampled from the cohort and used as the set of reference ‘normal’ cells. After subtracting out reference expression in non-cancer cells, chromosome-level smoothing and denoising with InferCNV, we derived a processed expression matrix that represented copy number signals. Cancer cell subclusters were identified by ward.D2 hierarchical clustering and the ‘random_trees’ partition method using *P* < 0.05.

##### Gene signature scores

Cell state scores were calculated for the exhausted phenotype within the set of T cells using a manually curated list of genes as input to the Seurat AddModuleScore method^40^. The curated list of genes was derived from a review of single-cell analyses of CD8^+^ T cell states in human cancers^41^ (Supplementary Table 4).

##### Patient specificity

Patient specificity scores were computed by using a shared nearest-neighbour graph. For a given cell, patient specificity was defined as the observed fraction of nearest neighbours divided by the expected fraction of nearest neighbours in the patient subgraph. Here the expected fraction of neighbours from the same patient was defined as the global fraction of cells for each patient. Scores were log_2_ transformed. Hence, a positive patient specificity score indicates an over-representation of cells derived from the same patient among its nearest neighbours, a negative score indicates an under-representation of cells from the same patient and a score of 0 reflects a perfectly mixed neighbourhood of patient labels.

##### Intra- and inter-patient variation in cluster composition

To calculate intra-sample diversity of cluster composition, we used the Shannon entropy *H*:

$$H=-\hspace{-.75mm}\mathop{\sum }\limits_{c=1}^{C}{p}_{c}\log \,{p}_{c}$$

where *p*_*c*_ is the proportional abundance of cluster *c* and *C* is the total number of clusters.

To estimate the similarity or dissimilarity between samples, we used the Bray–Curtis dissimilarity index *D* for samples *i* and *j*, defined as

$$D=1-\frac{2{\sum }_{c=1}^{C}{\rm{m}}{\rm{i}}{\rm{n}}({N}_{c}^{i},{N}_{c}^{j})}{{\sum }_{c=1}^{C}{N}_{c}^{i}+{\sum }_{c=1}^{C}{N}_{c}^{j}}$$

where ${N}_{c}^{i}$ and ${N}_{c}^{j}$ are the counts for cluster *c* in samples *i* and *j*, respectively, and *C* is the total number of clusters. This measure *D* takes values between 0 (identical samples: ${N}_{c}^{i}={N}_{c}^{j}$ for all *j*) and 1 (disjoint samples: ${N}_{c}^{i} > 0$ implies ${N}_{c}^{j}=0$). We only considered the triangular distance matrix *D* such that *i* < *j*. The pairwise distance matrix was estimated by randomly subsampling the dataset with a minimum number of cells per sample and averaging over the subsampled datasets after 100 iterations. We then evaluated intra- and inter-patient dissimilarity on the basis of the distributions of the off-diagonal elements in the averaged distance matrix (for example, all pairs of adnexal samples or all pairs of HRD-Dup samples).

These definitions were used to estimate the intra-sample diversity, intra-patient dissimilarity and inter-patient dissimilarity of cluster composition of cell states within each major cell type superset (cancer cells, Fig. 3g; T and NK cells, Figs. 2d and 4f; myeloid cells, Extended Data Figs. 6d and 11b). Rarefaction of samples was applied in estimation of the Bray–Curtis dissimilarity matrix on the basis of the number of cells for each subset (*n* = 400 cells per sample).

Finally, we also used non-metric multidimensional scaling (NMDS) to visualize the pairwise distances of cell type abundances in low-dimensional space. We used the pairwise dissimilarity matrix *D* to calculate the rank order of the Bray–Curtis distance and project differences in cluster composition in two dimensions using NMDS (cancer cells, Extended Data Fig. 8i; T and NK cells, Extended Data Figs. 7c and 10c; myeloid cells, Extended Data Figs. 7h and 11f).

##### GLMs of cluster composition

To estimate the effect of mutational signatures and tumour site specificity on the composition of cell clusters, we considered a GLM where we included interactions between signature, site and cluster identity for each major cell type defined in the scRNA-seq, H&E and mpIF data. The data matrix included the counts of every cluster *c*, sampled from site *s* in a patient with mutational signature subtype *m*. Using a binomial linear model, one can analyse counts of repeated observations of cell types or cell states as binary choices:

$${N}_{c} \sim {\rm{Bin}}({p}_{c},N)$$

where *N*_*c*_ is the cell count for cluster *c* in a sample, *N* is the total number of cells in the sample and the probability to detect the cluster can be described by the logit function $\log \frac{{p}_{c}}{1-{p}_{c}}=\beta X.$

To account for the effect of mutational signature and anatomical tumour site on the cluster abundance observed in scRNA-seq data, we formulated a GLM of the observed cell counts *N*_*c*_ for a cell type or cell state described by the logit function, which is distributed as

$$\log \,\frac{{p}_{c}}{1-{p}_{c}}\sim {\rm{N}}{\rm{o}}{\rm{r}}{\rm{m}}{\rm{a}}{\rm{l}}({\beta }_{0}+{\beta }_{c}{x}_{c}+{\beta }_{m}{x}_{m}+{\beta }_{s}{x}_{s}+{\beta }_{cm}{x}_{c}{x}_{m}+{\beta }_{cs}{x}_{c}{x}_{s},\,{\sigma }_{\varepsilon }^{2})$$

where *β*_0_ is a shared constant baseline per cluster that must be inferred; *β*_*c*_, *β*_*m*_ and *β*_*s*_ are individual fixed-effect terms to be inferred; *β*_*cm*_ and *β*_*cs*_ are cluster–signature and cluster–site interaction effects to be inferred; *x*_*c*_, *x*_*m*_ and *x*_*s*_ are elements of the model design matrix *X*; and *σ*_*ε*_ represents measurement noise. We note that for each cluster *c* we had multiple measurement replicates of *N*_*c*_ across signatures and sites. This formulation was used to fit a GLM of major cell types (Fig. 1f). We also used this formulation to separately fit GLMs of cluster composition for each superset of coarse-grained immune cell types (T and NK cells, Extended Data Fig. 7b; myeloid cells, Extended Data Figs. 7g and 11e) and GLMs of cluster composition for fine-grained immune cell states (T and NK cells, Fig. 2c; DCs, Extended Data Fig. 11a; macrophages, Extended Data Fig. 11a).

To model the abundance of major cell types in the scRNA-seq data from CD45^+^ and CD45^−^ samples, the GLM included a covariate for CD45^+/−^ flow sorting with additional fixed-effect sorting coefficients *β*_*f*_ and additional cluster sorting interactions *β*_*cf*_ to be inferred, plus an additional element *x*_*f*_ in the model design matrix (Fig. 1f). Similarly, GLMs for H&E and mpIF data accounted for differences in cell type abundance observed in the tumour and stroma regions, incorporating a covariate for the tumour or stroma region counts with additional fixed-effect region coefficients *β*_*r*_ and additional cluster–region coefficients *β*_*cr*_ to be inferred, plus an additional element *x*_*r*_ in the model design matrix (Fig. 1f).

To quantify interactions between mutational signature and anatomical tumour site, we also fitted GLMs with an additional interaction term:

$$\begin{array}{c}\log \frac{{p}_{c}}{1-{p}_{c}}\sim {\rm{N}}{\rm{o}}{\rm{r}}{\rm{m}}{\rm{a}}{\rm{l}}({\beta }_{0}+{\beta }_{c}{x}_{c}+{\beta }_{m}{x}_{m}+{\beta }_{s}{x}_{s}+{\beta }_{cm}{x}_{c}{x}_{m}\\ +{\beta }_{cs}{x}_{c}{x}_{s}+{\beta }_{csm}{x}_{c}{x}_{s}{x}_{m},\,{\sigma }_{\varepsilon }^{2})\end{array}$$

where *β*_*csm*_ terms were cluster-specific signature–site interaction effects to be inferred. This formulation was used to fit GLMs of cluster composition of cell states within each major cell type superset, both for fine-grained clusters (cancer cells, Fig. 3d; T and NK cells, Fig. 4b; DCs, Extended Data Fig. 11a; macrophages, Extended Data Fig. 11a) and coarse-grained clusters (T and NK cells, Extended Data Fig. 10e; myeloid cells, Extended Data Fig. 11e).

##### PD-1 and PD-L1/PD-L2 co-expression analysis

To determine potentially interacting cell type subclusters for the receptor-ligand pair PD-1–PD-L1/PD-L2, we first computed the fraction of sender cells (cancer cell or myeloid cell clusters) expressing the PD-L1 and PD-L2 ligands (*CD274* or *PDCD1LG2* read counts >0 in >10% of cells) and the fraction of receiver cells (T cell clusters) expressing the PD-1 receptor (*PDCD1* read counts >0 in >10% of cells) for every patient. Co-expression networks were constructed as follows: for a given group of patients of the same mutational subtype (Extended Data Fig. 13f), an edge was drawn between sender cell clusters and receiver cell clusters if the ligands (*CD274* or *PDCD1LG2*) and receptor (*PDCD1*) were co-expressed in the sender and receiver subclusters for at least 50% of patients in that group.

#### Bulk WGS

##### Alignment

Sequencing reads were aligned to human genome reference GRCh37 (hg19) using the Burrows–Wheeler aligner (BWA-MEM) v0.7.17-r1188 (https://sourceforge.net/projects/bio-bwa/).

##### Single-nucleotide variants and indels

Single-nucleotide variants (SNVs) and indels were called using mutationSeq (version 4.3.8; model v4.1.2.npz) available at https://github.com/shahcompbio/mutationseq. We also used Strelka (version 2.8.2) with default parameter settings to identify somatic SNVs and indels^42^. Both SNVs and indels were then annotated for variant effects and gene-coding status using SnpEff4 (version 5.0e). We identified a set of high-confidence SNVs by taking the intersection of the high-probability calls predicted from mutationSeq (with probability ≥0.9) and the somatic SNVs predicted by Strelka. The high-confidence set of SNVs was further filtered by removing positions that fell within either of the following regions: (1) the UCSC Genome Browser blacklists (Duke and DAC) and (2) regions defined in the ‘CRG Alignability 36mer track’ with more than two nucleotide mismatches, requiring a 36-nucleotide fragment to be unique in the genome even after allowing for two differing nucleotides. Postprocessing on this set of high-confidence SNVs and somatic indels from Strelka involved removing known variants (both SNVs and indels), which were obtained from the 1000 Genomes Project (release 20130502) and dbSNP (version dbsnp 142.human 9606). The set of high-confidence somatic SNVs and indels passing the above filters were then used in feature computation for mutational signature analysis and in neoantigen prediction.

##### Rearrangements

Rearrangement breakpoints were predicted using lumpy (version 0.2.12)^43^ executed by SpeedSeq (version 0.1.08)^44^ and destruct (version 0.4.18) derived from nFuse^45^, available at https://github.com/amcpherson/destruct. In brief, destruct extracted discordant and non-mapping reads from BAM files and realigned the reads using a seed-and-extend strategy. Split alignment across a putative breakpoint was attempted for reads that did not fully align to a single locus. Discordant alignments were clustered according to the likelihood that they were produced from the same breakpoint. Multiply mapped reads were assigned to a single mapping location using previously described methods^46^. Finally, heuristic filters removed predicted breakpoints with poor discordant read coverage of sequence flanking predicted breakpoints.

We applied stringent three-step filtering criteria to identify high-confidence breakpoint calls for downstream analysis as follows:

Step 1: Breakpoints that were predicted by both algorithms, lumpy and destruct, were taken forward.

Step 2: We removed (1) breakpoints from regions with poor mappability, (2) events with a break distance of ≤30 bp and (3) breakpoints annotated as a deletion with a breakpoint size of <1,000 bp. Furthermore, only high-confidence breakpoints that had at least five supporting reads in the tumour sample and no read support in the matched normal sample were used in the analysis. The breakpoints were further filtered by removing positions that fell in either of the following regions: (1) UCSC Genome Browser blacklists (Duke and DAC) and (2) regions defined in the ‘CRG Alignability 36mer track’ with more than two nucleotide mismatches, requiring a 36-nucleotide fragment to be unique in the genome even after allowing for two differing nucleotides.

Step 3: Predictions with a small break distance and a low number of supporting reads in tumour samples were excluded.

##### Copy number

Genome-wide allele-specific copy number was called in matched tumour–normal WGS samples using ReMixT^47^ and TitanCNA^48^ with default parameters. A parameter grid search for multiple purity and ploidy solutions was carried out, and the top solution was selected after manual assessment of the copy number segmentations. All tumour samples were run with ploidy = 2 and ploidy = 4 initializations.

#### Myriad HRD test

We used a commercial assay (Myriad Genetics ‘myChoice CDx’) to test for genome-wide LOH, the number of chromosomal breakpoints in large-scale state transitions and telomeric allelic imbalance. If the resulting HRD score was greater than 42, the sample was deemed to be HRD.

#### Targeted sequencing (MSK-IMPACT)

Genomic DNA isolated from FFPE tumour tissue and matched normal blood was subjected to hybridization capture and sequenced with deep coverage (700×)^49^. Variant calling for the MSK-IMPACT gene panel and copy number analysis were performed using the MSK-IMPACT clinical pipeline (https://github.com/mskcc/Innovation-IMPACT-Pipeline).

#### Mutational signatures

We analysed mutational signatures by integrating SNVs and structural variations detected by bulk WGS in a unified probabilistic approach called multimodal correlated topic models (MMCTM)^6^. MMCTM analysis enables robust determination of mutational signatures and their correlation structure and delineation of subgroupings based on point mutation signatures^50^ and structural variations.

We estimated signature probabilities for bulk WGS samples in the MSK SPECTRUM cohort (*n* = 40) using MMCTM, on the basis of SNV and structural variation signatures inferred from HGSOC (*n* = 170) and triple-negative breast cancer (*n* = 139) bulk whole genomes (total *n* = 309) (Extended Data Fig. 2b). By clustering the meta-cohort of 309 HGSOC and triple-negative breast cancer samples using UMAP and HDBSCAN^51^, we used the meta-cohort as a training dataset to fit a *k*-nearest-neighbour (kNN) classifier and applied the kNN classifier to the SPECTRUM samples (*n* = 40), assigning them into one of four strata defined solely by SNV and structural variation signature probabilities. A nearest-neighbour graph was built using a Euclidean distance metric, and classification into strata was computed by a majority vote of the *k* nearest neighbours of the unknown test sample (*k* = 30), requiring *m* votes for an assignment (*m* = 25). The four strata included those with samples enriched for (1) *BRCA1*-associated HRD point mutation signatures accompanied by tandem duplications (HRD-Dup), (2) *BRCA2*-associated HRD point mutation signatures accompanied by interstitial deletions (HRD-Del), (3) foldback inversions mediated by breakage–fusion bridge cycles (FBI) and (4) a group of ambiguous samples near the classifier decision boundaries (‘Undetermined’) (Extended Data Fig. 2c).

To validate the MMCTM mutational signatures, we used two independent computational methods (Extended Data Fig. 2b). We applied HRDetect^18^ to validate HRD status on the basis of SNV signatures previously associated with HRD (SBS3, SBS8), short microhomology-mediated indels (ID8) and rearrangement signatures (RS3, RS5). Samples with an HRDetect score of >0.1 were defined as HRD. We also applied CHORD^19^ to validate HRD status and stratification of HRD-Dup from HRD-Del cases. CHORD incorporates SNVs, indels and structural variations and relies on duplications (1–100 kb) to distinguish *BRCA1*-like from *BRCA2*-like HRD.

WGS-derived HRD signatures were in agreement for seven of seven cases with *BRCA1* or *BRCA2* loss (Extended Data Fig. 2b). WGS-based and standard-of-care HRD status were concordant in five of six cases. The discordant case (024) was deemed HRD by all three independent methods for WGS signature inference (MMCTM, HRDetect and CHORD).

#### Focal amplifications and deletions

We used WGS copy number inferred by ReMixT^47^ to classify copy number changes as focal amplifications and deletions in the MSK SPECTRUM cohort. For focal amplifications, we calculated the percentile of each gene with respect to the cumulative distribution of total copy number changes across the genome. On the basis of the mean copy number across each gene, we classified high-level amplifications as those in the top 2% of bins with a log_2_-transformed change over ploidy greater than 1. For homozygous deletions, we considered gene copy number in overlapping segments and we classified segments that were 10 kb or greater in size with a mean copy number less than 0.5 as homozygous deletions.

Similarly, we used IMPACT copy number inferred by FACETS^52^ to delineate focal amplifications and homozygous deletions in the MSK IMPACT HGSOC cohort. Focal amplifications and deletions were identified on the basis of the median copy number log ratio per segment, only considering segments shorter than 10 Mb with ten or fewer genes to suppress arm-level events. Segments with a total copy number greater than 8 were considered as high-level amplifications. Homozygous deletions were called for segments with a total copy number of 0.

#### HLA LOH

To detect allele-specific copy number LOH of the HLA locus in single cells profiled by scRNA-seq, we inferred allele-specific alterations on chromosome arm 6p, which harbours HLA class I and II genes, using SIGNALS^5^. We first called germline heterozygous single-nucleotide polymorphisms (SNPs) in the scRNA-seq tumour data using cellSNP^53^. As input, we used the set of heterozygous SNPs identified in the corresponding normal WGS dataset for each sample. The liftover script provided in cellSNP was used to lift over SNP coordinates from the GRCh37 (hg19) reference genome to the GRCh38 reference genome. Following genotyping, we aggregated SNP counts across all cells and defined the B allele as the allele with the lowest allele frequency for each SNP. As SNP counts are very sparse in scRNA-seq data, we then aggregated cell-level counts of the B allele across chromosome arms to compute the BAF for each arm in each cell. We then generated a cell-by-chromosome arm BAF matrix and incorporated this into the Seurat gene expression objects. To assign allelic imbalance states (balanced, imbalanced, LOH) to chromosome arms in each cell, we used the mean BAF of each arm per cell as follows: balanced, BAF ≥ 0.35; imbalanced, 0.15 ≤ BAF < 0.35; LOH, BAF < 0.15. Documentation and code are available at https://shahcompbio.github.io/signals/.

To validate our observations of allele-specific alterations on chromosome arm 6p in relation to the HLA locus, we detected gene-level HLA class I LOH from tumour and matched normal WGS data, as well as from tumour and matched normal MSK-IMPACT data, using LOHHLA^24^.

To validate HLA LOH status by WGS, we used 40 tumour–normal pairs from 40 patients. Tumour purity and ploidy were estimated using ReMiXT^47^ and used for subsequent HLA LOH analysis. To validate HLA LOH status by MSK-IMPACT, we selected 1,298 tumour–normal pairs from 1,298 patients in the MSK-IMPACT cohort with HGSOC histology based on an HGSOC or HGSFT OncoTree classification^54^. This cohort did not include MSK-IMPACT samples from patients who were part of the MSK SPECTRUM cohort.

Patient HLA references were built from tumour and normal reads using Polysolver (v4)^55^, for both WGS and MSK-IMPACT data. Tumour purity and ploidy from the WGS datasets were estimated using ReMixT^47^ and used for subsequent HLA LOH analysis. Similarly, tumour purity and ploidy for the MSK-IMPACT datasets were estimated using FACETS^52^. HLA LOH was called for an allele in the tumour sample using LOHHLA. LOH was observed for each HLA gene if the estimated copy number was <0.2 and the statistical significance of the allelic imbalance was *P* < 0.01, testing for pairwise differences in log(*R*) values between the two HLA homologues (paired *t* test).

#### Digital histopathology

We built a training dataset of cellular annotations for scanned H&E images. Expert delineation and quantification of cell and tissue types present in the H&E slides was carried out on MSK Slide Viewer, a computational pathology interface for review and annotation of histopathology images. Nuclear segmentation was carried out using StarDist, a method for nuclear detection based on the U-Net neural network architecture^56,57^. Membrane segmentation was approximated using a cell expansion of 3 μm of the nuclear boundary. The training dataset encompasses a set of 61 slides from a representative set of patients and sites. To classify regions of tumour, stroma, vasculature and necrosis, we trained an artificial neural network (ANN)-based pixel classifier using QuPath (v0.2.3)^56^, which operates on higher-order pixel features over multiple channels and scales within an image. In addition, lymphocytes and ‘other’ cells were annotated in 19 of these slides by a researcher using MSK Slide Viewer. After importing these annotations into QuPath, along with cellular segmentations and feature vectors generated from StarDist, we trained an ANN-based cellular classifier that operates over cellular measurements to identify lymphocytes. We then applied these models for inference across 100 whole-slide H&E images from 35 patients. Segmentation yielded a total of 24,628,462 cells across samples, and we used the model outputs to compute statistics on lymphocyte densities and other spatially derived measurements.

#### mpIF

We carried out nuclear segmentation based on DAPI intensity using the watershed algorithm in QuPath (v0.2.3)^56^, setting a minimum DAPI threshold of 1 arbitrary unit with an expected nucleus area ranging between 5 μm^2^ and 100 μm^2^. Membrane segmentation was approximated using a cell expansion of 3 μm of the nuclear boundary. Starting from 1,349 quality-filtered FOVs across 100 tissue samples from 35 patients, segmentation yielded a total of 10,892,612 cells. To annotate regions of tumour and stroma, we trained a pixel classifier with examples of panCK^+^ (tumour) and panCK^−^ (stroma) regions. Following nuclear segmentation, we extracted the pixel intensities per cell for functional markers expressed in the cytoplasm (panCK, CD68, CD8, PD-1, PD-L1) and in the nucleus (TOX) to define cell types and cell states. All channels were manually thresholded in at least one FOV per slide, and marker positivity was determined by setting these thresholds on the mean pixel intensity. Segmented objects that were double or triple positive for multiple cell type markers (panCK, CD68, CD8) were counted as separate cells, yielding a total of 12,359,463 single cells. Marker assignments were used to define cell states of epithelial cells (panCK^+^PD-L1^−^, panCK^+^PD-L1^+^), macrophages (CD68^+^PD-L1^−^, CD68^+^PD-L1^+^) and CD8^+^ T cells (CD8^+^PD-1^−^TOX^−^, CD8^+^PD-1^+^TOX^−^, CD8^+^PD-1^+^TOX^+^).

Analysis of spatial topology comprised estimation of spatial densities and intercellular nearest-neighbour distances. Spatial density estimates as a function of distance to the tumour–stroma boundary were obtained by aggregating cell counts within 10 μm distance bands from the boundary in each FOV, grouped across FOVs and normalized by the total number of cells for a given phenotype of interest. Error bars were calculated as the standard error of the probability *p* of observing a given phenotype as $\sqrt{p(1-p)/N}$, where *N* was the total number of cells in the distance band. Intercellular distances between nearest neighbours were calculated using the distance matrix *r*_*ij*_ for cells *i* and *j*, where the value of the (*i*, *j*) element in the matrix was the radial distance from cell *i* to cell *j*. After computing per-cell nearest neighbours, the summary statistics over nearest-neighbour distances were estimated for each phenotype. Proximity counts for phenotypes within a fixed radius *R* were also determined on the basis of per-cell nearest neighbours.

### Reporting summary

Further information on research design is available in the Nature Portfolio Reporting Summary linked to this article.

## Online content

Any methods, additional references, Nature Portfolio reporting summaries, source data, extended data, supplementary information, acknowledgements, peer review information; details of author contributions and competing interests; and statements of data and code availability are available at 10.1038/s41586-022-05496-1.

### Supplementary information

Reporting Summary

Supplementary Table 1 Clinical overview of the MSK SPECTRUM patient cohort. References to validation cohorts (MSK IMPACT HGSOC cohort and HGSOC/TNBC meta-cohort).

Supplementary Table 2 Sample inventory of the MSK SPECTRUM patient cohort. Metadata associated with scRNA-seq, H&E, mpIF, bulk tumour and normal WGS, Myriad HRD tests and tumour and normal MSK-IMPACT datasets.

Supplementary Table 3 Mutational signature proportions and mutational subtype assignments from WGS datasets for MSK SPECTRUM patients.

Supplementary Table 4 Cell type and cell subtype markers. Clusters were annotated on the basis of marker genes identified in differential gene expression analysis of scRNA-seq data.

Supplementary Table 5 GLMs for major cell types, cancer cells, T/NK cells, DCs and macrophages.

Supplementary Table 6 Antibodies and staining conditions for mpIF.

## Data Availability

Datasets generated and analysed in this study are available for general research use and are documented in Synapse (https://www.synapse.org/msk_spectrum). Open-tier datasets not requiring access approval are available for download via Synapse (accession number syn25569736: https://www.synapse.org/msk_spectrum). Controlled-tier datasets requiring access approval are available by requesting authorisation to the Data Access Committee via dbGaP (accession number phs002857.v1.p1: http://www.ncbi.nlm.nih.gov/projects/gap/cgi-bin/study.cgi?study_id=phs002857.v1.p1). An interactive visualization interface for the scRNA-seq data from this study is available via CELLxGENE (https://cellxgene.cziscience.com/collections/4796c91c-9d8f-4692-be43-347b1727f9d8). The WGS and MSK-IMPACT datasets are available for browsing via cBioPortal (https://www.cbioportal.org/study/summary?id=msk_spectrum_tme_2022).

## Code availability

The code is publicly accessible in a GitHub repository (https://github.com/shahcompbio/spectrum-tme), which describes how to reproduce the figures and tables in this publication.
