# Supplementary material for: Ovarian cancer mutational processes drive site-specific immune evasion
Source: Nature. 2022 Dec 14;612(7941):778–86. doi: 10.1038/s41586-022-05496-1 (PMC9771812; doi:10.1038/s41586-022-05496-1)
Supplement: Supplementary file 1 — Reporting Summary [file 41586_2022_5496_MOESM1_ESM.pdf]

nature portfolio

Corresponding author(s): Sohrab P. Shah, Dmitriy Zamarin

Last updated by author(s): Dec 1, 2022

Reporting Summary

Nature Portfolio wishes to improve the reproducibility of the work that we publish. This form provides structure for consistency and transparency in reporting. For further information on Nature Portfolio policies, see our Editorial Policies and the Editorial Policy Checklist.

Statistics

For all statistical analyses, confirm that the following items are present in the figure legend, table legend, main text, or Methods section.

n/a	Confirmed	
☐	☒	The exact sample size (*n*) for each experimental group/condition, given as a discrete number and unit of measurement
☐	☒	A statement on whether measurements were taken from distinct samples or whether the same sample was measured repeatedly
☐	☒	The statistical test(s) used AND whether they are one- or two-sided<br>*Only common tests should be described solely by name; describe more complex techniques in the Methods section.*
☐	☒	A description of all covariates tested
☐	☒	A description of any assumptions or corrections, such as tests of normality and adjustment for multiple comparisons
☐	☒	A full description of the statistical parameters including central tendency (e.g. means) or other basic estimates (e.g. regression coefficient) AND variation (e.g. standard deviation) or associated estimates of uncertainty (e.g. confidence intervals)
☐	☒	For null hypothesis testing, the test statistic (e.g. *F*, *t*, *r*) with confidence intervals, effect sizes, degrees of freedom and *P* value noted<br>*Give P values as exact values whenever suitable.*
☒	☐	For Bayesian analysis, information on the choice of priors and Markov chain Monte Carlo settings
☐	☒	For hierarchical and complex designs, identification of the appropriate level for tests and full reporting of outcomes
☐	☒	Estimates of effect sizes (e.g. Cohen's *d*, Pearson's *r*), indicating how they were calculated

Our web collection on statistics for biologists contains articles on many of the points above.

Software and code

Policy information about availability of computer code

Data collection	scRNA-seq pipeline: https://github.com/shahcompbio/scrna-pipeline<br>WGS pipeline: https://github.com/shahcompbio/wgs
Data analysis	Figures and tables: https://github.com/shahcompbio/spectrum-tme

For manuscripts utilizing custom algorithms or software that are central to the research but not yet described in published literature, software must be made available to editors and reviewers. We strongly encourage code deposition in a community repository (e.g. GitHub). See the Nature Portfolio guidelines for submitting code & software for further information.

Data

Policy information about availability of data

All manuscripts must include a data availability statement. This statement should provide the following information, where applicable:

- Accession codes, unique identifiers, or web links for publicly available datasets
- A description of any restrictions on data availability
- For clinical datasets or third party data, please ensure that the statement adheres to our policy

The MSK SPECTRUM study is registered on dbGaP under accession number phs002857.v1.p1 (https://www.ncbi.nlm.nih.gov/projects/gap/cgi-bin/study.cgi?study_id=phs002857.v1.p1).

A Synapse page for the MSK SPECTRUM study is available to provide access to multi-modal datasets from one central location. This page can be accessed under accession number syn25569736 (https://www.synapse.org/msk_spectrum).

Data availability:
• scRNA-seq:
- Raw and processed expression data are available from NCBI Gene Expression Omnibus (https://www.ncbi.nlm.nih.gov/geo/query/acc.cgi?acc=GSE180661).
- Processed objects are available from Synapse (https://www.synapse.org/#!Synapse:syn33521743/datasets/).
- Interactive data visualizations are available via CELLxGENE (https://cellxgene.cziscience.com/collections/4796c91c-9d8f-4692-be43-347b1727f9d8).
• Tumour-normal bulk WGS:
- Raw sequencing reads are available for controlled access from the NCBI Sequence Read Archive via dbGaP (https://www.ncbi.nlm.nih.gov/projects/gap/cgi-bin/study.cgi?study_id=phs002857.v1.p1).
- Somatic mutations and copy number data can be accessed from Synapse (https://www.synapse.org/#!Synapse:syn33521770/datasets/).
- Somatic mutations and copy number can be visualized through cBioPortal (https://cbioportal.org/study/summary?id=msk_spectrum_tme_2022).
• Tumour-normal targeted panel sequencing (MSK-IMPACT):
- Somatic mutations and copy number can be visualized through cBioPortal (https://cbioportal.org/study/summary?id=msk_spectrum_tme_2022).
• H&E:
- Deidentified images are available via dbGaP (https://www.ncbi.nlm.nih.gov/projects/gap/cgi-bin/study.cgi?study_id=phs002857.v1.p1).
- Cell segmentation, tissue segmentation and lymphocyte classification are available from Synapse (https://www.synapse.org/#!Synapse:syn33521762/datasets/).
• mpIF:
- Deidentified images are available via dbGaP (https://www.ncbi.nlm.nih.gov/projects/gap/cgi-bin/study.cgi?study_id=phs002857.v1.p1).
- Cell segmentation, tissue segmentation and cell phenotyping are available from Synapse (https://www.synapse.org/#!Synapse:syn33520881/datasets/).

Field-specific reporting

Please select the one below that is the best fit for your research. If you are not sure, read the appropriate sections before making your selection.

☒ Life sciences ☐ Behavioural & social sciences ☐ Ecological, evolutionary & environmental sciences

For a reference copy of the document with all sections, see nature.com/documents/nr-reporting-summary-flat.pdf

Life sciences study design

All studies must disclose on these points even when the disclosure is negative.

Sample size	Quality-filtered study datasets (MSK SPECTRUM cohort):<br>• scRNA-seq: 41 patients, 156 samples, 929,686 cells<br>• H&E: 35 patients, 100 samples, 24,628,462 cells<br>• mpIF: 35 patients, 100 samples, 1,349 fields of view, 12,359,463 cells<br>• Bulk tumour WGS: 40 patients, 40 samples<br>• Bulk normal WGS: 42 patients, 42 samples<br>• Myriad HRD test: 9 patients, 9 samples<br>• Tumour-normal targeted panel sequencing (MSK-IMPACT): 42 patients, 42 samples<br><br>Validation dataset (MSK IMPACT HGSOC cohort):<br>• Tumour-normal targeted panel sequencing (MSK-IMPACT): 1,298 patients, 1,298 samples
Data exclusions	Low-quality cells were removed from scRNA-seq analyses, and fields of view with scant tissue were removed from mpIF analyses, as described in the methods.
Replication	Patients with multi-site data had 3-4 samples profiled by scRNA-seq, H&E and mpIF. Associations of immune and malignant cell phenotypes with signature- and site-specific covariates were concordant and could be validated by scRNA-seq, H&E and mpIF data. Genomic profiling was also consistent between bulk WGS, Myriad HRD tests and targeted panel sequencing (MSK-IMPACT).
Randomization	Patients were stratified into groups based on mutation signature type, as described in the manuscript.
Blinding	Group allocation was done by assigning patients to mutational signature types based on data analysis described in the manuscript.

Reporting for specific materials, systems and methods

We require information from authors about some types of materials, experimental systems and methods used in many studies. Here, indicate whether each material, system or method listed is relevant to your study. If you are not sure if a list item applies to your research, read the appropriate section before selecting a response.

Materials & experimental systems

n/a	Involved in the study	
☐	☒	Antibodies
☒	☐	Eukaryotic cell lines
☒	☐	Palaeontology and archaeology
☒	☐	Animals and other organisms
☐	☒	Human research participants
☐	☒	Clinical data
☒	☐	Dual use research of concern

Methods

n/a	Involved in the study	
☒	☐	ChIP-seq
☐	☒	Flow cytometry
☒	☐	MRI-based neuroimaging

Antibodies

Antibodies used

Antigen	Antibody Clone	Manufacturer	Titration	Detection Dye (cycle)
CD68	D4B9C	Cell Signaling	0.0486 μg/ml	Opal 520 (1)
TOX	E6I3Q	Cell Signaling	0.0644 μg/ml	Opal 540 (2)
PD-1	EPR4877(2)	Abcam	5 μg/ml	Opal 570 (3)
PD-L1	73-10	Abcam	0.18 μg/ml	Opal 620 (4)
CD8	C8/144B	Cell Signaling	0.125 μg/ml	Opal 650 (5)
panCK	AE1/AE3	Dako	0.665 μg/ml	Opal 690 (6)
CK8/18	C51	Cell Signaling	0.16 μg/ml	Opal 690 (6)

Validation

Primary antibody staining conditions were optimized using standard immunohistochemical staining on the Leica Bond RX automated research stainer with DAB detection (Leica Bond Polymer Refine Detection DS9800). Using 4 μm formalin-fixed, paraffin-embedded tissue sections and serial antibody titrations, the optimal antibody concentration was determined followed by transition to a seven-color multiplex assay with equivalency. Optimal primary antibody stripping conditions between rounds in the seven-color assay were performed following 1 cycle of tyramide deposition followed by heat-induced stripping (see Methods) and subsequent chromogenic development (Leica Bond Polymer Regine Detection DS9800) with visual inspection for chromogenic product with a light microscope by a senior pathologist.

Human research participants

Policy information about studies involving human research participants

Population characteristics

The study cohort (MSK SPECTRUM) includes 42 women with newly diagnosed, treatment-naive high-grade serous ovarian cancer (HGSOC). Patients between the ages of 39 and 81 at diagnosis (median age: 61 years). 6 out of 42 cases had BRCA1 mutations (14%) and 1 out of 42 cases had a BRCA2 mutation (2%).

Recruitment

All enrolled patients were consented to an institutional biospecimen banking protocol and a protocol to perform targetted panel sequencing (MSK-IMPACT). All analyses were performed per a biospecimen research protocol. All protocols were approved by the Institutional Review Board (IRB) of Memorial Sloan Kettering Cancer Center (MSKCC). Patients were consented following the IRB-approved standard operating procedures for informed consent. Written informed consent was obtained from all patients before conducting any study-related procedures. This study was conducted in accordance with the Declaration of Helsinki and the Good Clinical Practice guidelines (GCP).

Ethics oversight

Institutional Review Board (IRB) at Memorial Sloan Kettering Cancer Center (MSKCC).

Note that full information on the approval of the study protocol must also be provided in the manuscript.

Clinical data

Policy information about clinical studies

All manuscripts should comply with the ICMJE guidelines for publication of clinical research and a completed CONSORT checklist must be included with all submissions.

Clinical trial registration

Not applicable.

Study protocol

The clinical study was conducted under the purview of MSKCC institutional tissue banking protocol 06-107 titled "Storage and research use of human biospecimens". Data generation and data analysis were carried out under MSKCC protocol 15-200 titled "Chemotherapy, somatic mutations, neoantigens, and the immune environment in ovarian cancer". Protocol 15-200 operates by using specimens banked under protocol 06-107. Due to MSKCC standard operating policies, internal protocol documents cannot be shared publicly but are available upon request to the IRB.

Data collection

Patients were enrolled on the study at MSKCC in New York, USA. Sample collection took place between January 2019 and March 2021.

Outcomes

No patient outcomes were reported.

March 2021

Flow Cytometry

Plots

Confirm that:

- [ ] The axis labels state the marker and fluorochrome used (e.g. CD4-FITC).
- [ ] The axis scales are clearly visible. Include numbers along axes only for bottom left plot of group (a 'group' is an analysis of identical markers).
- [ ] All plots are contour plots with outliers or pseudocolor plots.
- [ ] A numerical value for number of cells or percentage (with statistics) is provided.

Methodology

Sample preparation	Freshly dissociated cells were stained with a mixture of GhostRed780 live/dead marker (TonBo Biosciences) and Human TruStain FcX™ Fc Receptor Blocking Solution (BioLegend). The stained samples were then incubated and stained with Alexa Fluor® 700 anti-human CD45 Antibody (BioLegend). Post staining, they were washed and resuspended in RPMI + 2% FCS and submitted for cell sorting. The cells were sorted into CD45 positive and negative fractions by fluorescence assisted cell sorting (FACS) on a BD FACSAria™ III flow cytometer (BD Biosciences). Positive and negative controls were prepared and used to set up compensations on the flow cytometer. Cells were sorted into tubes containing RPMI + 2% FCS for sequencing.
Instrument	BD FACSAria™ III
Software	BD FACSADiva™ Software
Cell population abundance	After gating for live cells, the fraction of CD45+ and CD45- cells was determined out of the total number of live cells.
Gating strategy	Preliminary FSC/SSC gating of live cells was followed by gating of CD45+ and CD45- populations of live cells.

- [x] Tick this box to confirm that a figure exemplifying the gating strategy is provided in the Supplementary Information.
